# Supplementary material for: Impact of enterprise digital transformation on capacity utilization: Evidence from China
Source: PLoS One. 2023 Mar 17;18(3):e0283249. doi: 10.1371/journal.pone.0283249 (PMC10022773; doi:10.1371/journal.pone.0283249)
Supplement: Attachment — Submitted filename: Referee Report_PONE-D-22-19462.pdf [file pone.0283249.s002.pdf]

Referee report: “Enterprise Digital Transformation and Capacity Utilization.” (PONE-D-22-19462)

1 Summary

This paper studies the impact of digitalization on the capacity utilization of firms through the path of collaborative relationships and efficient innovations. Through literature reviews and qualitative analysis, the author abstracts three core hypotheses and empirical studies using the data from A-share listed companies in China find that the impact mechanism of firms’ digital transformation on its capacity utilization is nonlinear U-shaped. Endogenous analysis, robust analysis and heterogeneous analysis further confirm this conclusion.

2 Main comments

1. The English writing of this article is not up to standard, there are many grammatical errors, and the expression is not authentic, which is far from meeting the requirements of publication. There are unnecessary long difficult sentences in the article, and too many “and” are inappropriately used. I suggest the author(s) carefully revise the paper and submit it to a Chinese journal; otherwise the language should be greatly improved.

2. Many of the discussions and conclusions in this paper are neither supported by theoretical literature nor supported by theoretical model analysis, which greatly affects the credibility of this paper, e.g. the paper concludes that “digital transformation of enterprise will promote innovation, improve resource allocation and production capacity, so as to effectively curb overcapacity”. How to curb overcapacity through digital transformation is an interesting question, but there is no model or theoretical analysis in this paper, so the mechanism is not well explored. As a result, many conclusions are just like basic judgments. I suggest the authors write a model to explain how digital transformation can better curb overcapacity compared with other ways.

3. Three hypotheses are not well organized. As far as I’m concerned, H1, H2 and H3 are about a same thing: how digitalization promotes capacity utilization, so the main topic can be summarized into one hypothesis, which can be treated as the paper’s main proposition and contribution. As stated in point 2, the authors can write a theoretical model to see whether the U-shaped nonlinear relationship can be derived from the model.

3 Minor comments and typos

1. The authors cite so many Chinese journals, and there are no more than 8 articles cited from international authoritative journals.

2. The citing format in the article is wrong.

3. The formula is not fully displayed, although I can guess what it is.
