# Supplementary material for: Impact of enterprise digital transformation on capacity utilization: Evidence from China
Source: PLoS One. 2023 Mar 17;18(3):e0283249. doi: 10.1371/journal.pone.0283249 (PMC10022773; doi:10.1371/journal.pone.0283249)
Supplement: Attachment — Submitted filename: PONE-D-22-19462.pdf [file pone.0283249.s004.pdf]

PLOS ONE

Impact of enterprise digital transformation on capacity utilization: Evidence from China

--Manuscript Draft--

**Manuscript Number:**	PONE-D-22-19462R1
**Article Type:**	Research Article
**Full Title:**	Impact of enterprise digital transformation on capacity utilization: Evidence from China
**Short Title:**	Impact of enterprise digital transformation on capacity utilization
**Corresponding Author:**	Ning Zhao<br>Zhongnan University of Economics and Law - Nanhu Campus: Zhongnan University of Economics and Law<br>Wuhan, CHINA
**Keywords:**	Digital Transformation; vertical specialization; enterprise innovation; capacity utilization rate
**Abstract:**	This study analyzes data from Chinese listed companies from 2010 to 2018, and demonstrates that enterprises' digital transformation has a significant impact on increasing the rate of capacity utilization,and there is an inverted U-shaped relationship. Digital transformation, in particular, is a significant driving force of enterprise-specific production and innovation. Furthermore, enterprise innovation and enterprise-specialized production play a mediating role in the impact of enterprise digital transformation on capacity utilization. Further analysis of heterogeneity reveals that the impact of digital transformation on capacity utilization is significant for firms with larger capital scales or with poor governance and manufacturing. However, it is less important in enterprises with small and medium capital scales or with more standardized governance, as well as in non-manufacturing (service) enterprises. In summary, this study investigates the mechanisms and economic impact of digital transformation, and its findings have critical theoretical and practical implications.
**Order of Authors:**	Ning Zhao
	Jianxin Ren
**Response to Reviewers:**	Dear reviewers,<br>Thank you so much for your comments and professional advice. These comments helped to improve the academics of my article. Following your suggestions and requests, we have made corrections to the revised manuscript. At the same time, the grammar of the manuscript has been reviewed and edited. I hope our work can be improved again. Moreover, we would like to show the details as follows:<br><br>Reviewer 1 #<br>1- There are a huge number of published studies available in the field. How this study significantly contributes to the literature?<br>The author's answer: The reviewer makes a valid point that there are numerous articles on digital transformation and a relatively large number of articles studying capacity, but almost no articles examining the effect of digital transformation on capacity utilization. Therefore, this study opens up a contemporary research perspective on digital transformation. Moreover, most of the studies on digital transformation have been conducted from a positive perspective to investigate its impact mechanisms. Based on this, the research presented in this paper adds a study on the negative effects of digital transformation to fill the gap in research thinking. In addition, current measurements and characterization of capacity utilization are usually based on a macro level, e.g., country, region, etc., whereas the calculations of capacity utilization in this paper are at a more microscopic level, which leads to more accurate conclusions. (See I. Introduction in the revised manuscript for details)<br><br>2-Please cite papers from the different areas, as the author mainly focuses on Chinese literature such as "China has made significant achievements in economic growth". While many countries, such as India, the USA, and Israel, are adopting digital

transformation.
The author’s answer: The reviewer’s comments are professional. With the development of science and technology, digital transformation is advocated in many countries, and there are many geographically distributed companies actively implementing digital management and services. However, there are some fundamental differences between these countries and regions and Chinese companies, such as over-building, over-investment, which hindered the efficient use of their resources and even prevents healthy economic development, and low capacity utilization and over-capacity. On top of this, the Chinese government urgently needs to find a tool to reverse this passive situation, so the digital transformation of local companies is particularly important. For these reasons, it is a more appropriate choice to study the impact of digital transformation on capacity ratios by analyzing the mechanism of its effect using data from Chinese-listed companies.

3-Expand the introduction by supporting the difference between digital and conventional transformation and cite some high-quality papers to support the idea.
The author’s answer: Your advice is perfectly professional. Based on your comments, I have added a high-level article related to the topic in the Introduction section as appropriate, as well as a more in-depth analysis and categorization of the current research on digital transformation. I have identified the current research gap in the analysis of the role of digitalization mechanisms and explored them as a research focus.

4- Scientific literature is lacking, please include more references related to the study.
The author’s answer: Thank you for your corrections. It has been added.

5- There are many characteristics of the overcapacity industry, but I can see only three have been mentioned in section II.
The author’s answer: Thank you for the correction. Excess capacity may exist at different stages of industry development. However, the causes of overcapacity are very different. For example, overcapacity is caused by competition in the early stages of industry development, or overcapacity is caused by declining market demand in sunset industries. Since this is not the focus of this paper, I have not highlighted it in the paper after further consideration.

6- Section II, First Paragraph, what do the distinctive Chinese characteristic please specify, and explain how these characteristics result in overcapacity.
The author’s answer: Thank you for the reminder, and based on your suggestion, I have added an introduction to Chinese firm features in the first section. The term capacity utilization, which serves as the focal explanatory variable in this study, has not been prominent in other country studies. This may be related to the historical process of overcapacity formation. It is well known that the most direct cause affecting capacity utilization is over-investment, and there are many causes that induce over-investment. In compiling the literature, I found that the main factors contributing to overcapacity in academia are broadly divided into three categories: 1. Industry competition, excessive competition in the industry, competitive strategies adopted by enterprises to deter and threaten their competitors, which triggers the expansion of production capacity and induces overcapacity; 2. Development dynamics, the frequent occurrence of investment surges in developing countries, and the role of market interest rates and price adjustment mechanisms are emphasized in the theory. The theory is that in developing countries, a large influx of capital into certain industries, i.e., an "investment boom", triggers the phenomenon of increasing overcapacity; 3. Government-business relationship, the most commonly studied theory in China, argues that local officials want to provide land and financing investments to local enterprises in the promotion race, resulting in the over-expansion of capacity and causing under-utilization of capacity.

7- Section II first paragraph, “The 2022 Government work report” cite the reference.
The author’s answer: Sorry for this error due to my negligence. Thank you for pointing it out, it has been corrected.

8- Please include the references to support your result and conclusion. Interpretation needs to be simplified and discussed with references.
The author’s answer: Thank you so much for your suggestion. I have made changes.

Powered by Editorial Manager® and ProduXion Manager® from Aries Systems Corporation

9- Include the limitation of the study.
The author’s answer: In light of your comments and suggestions, we have added the limitations of the study in the "1 Introduction" section.

10- There are many typing and grammatical errors, which needs to be corrected.
The author’s answer: Thank you for your advice. The grammatical problems have been corrected one by one.

Reviewer 2 #
Main comments
1.The English writing of this article is not up to standard, there are many grammatical errors, and the expression is not authentic, which is far from meeting the requirements of publication. There are unnecessary long difficult sentences in the article, and too many “and” are inappropriately used. I suggest the author(s) carefully revise the paper and submit it to a Chinese journal; otherwise the language should be greatly improved.
The author’s answer: Thank you for your criticism. I re-read the article carefully and revised it, and have made sufficient repairs to eliminate grammatical errors, correct the use of "and", and make changes to the long and difficult sentences.

2.Many of the discussions and conclusions in this paper are neither supported by theoretical literature nor supported by theoretical model analysis, which greatly affects the credibility of this paper, e.g. the paper concludes that “digital transformation of enterprise will promote innovation, improve resource allocation and production capacity, so as to effectively curb overcapacity”. How to curb overcapacity through digital transformation is an interesting question, but there is no model or theoretical analysis in this paper, so the mechanism is not well explored. As a result,many conclusions are just like basic judgments. I suggest the authors write a model to explain how digital transformation can better curb overcapacity compared with other ways.
The author’s answer: The reviewer makes very good points and your suggestions are very professional. The logic and theory of the article have been further refined and reconstructed with your suggestions. In the introduction section, I appropriately introduce the many high-quality works in the literature to sort out the objective factors the influence capacity utilization at a more specific level, as well as to point out the reasons for selecting Chinese-listed companies as the target of the study. In addition, in sorting out the impact and mechanism of action of current enterprise capacity utilization, I discovered its research gap - on capacity utilization, which provides ideas for further analysis and paves the way for the theoretical analysis and research hypothesis in the later sections.
In the theoretical analysis sections, I adopt the previous analysis framework and introduce the main assumptions of this paper based on reading a large amount of high-level literature. The theoretical analysis is taken from the classical literature, which enhances the credibility of the article. In the analysis of the impact of the digital transformation of firms on capacity utilization, two mediating variables, firm innovation, and specialized production, are introduced in this paper. Among them, for the hypothesis that digital transformation drives enterprise innovation, I explore in more depth the specific paths and implementation capabilities of innovation affecting enterprise capacity by specifically analyzing the factors that affect the role of capacity utilization and enumerating the mechanisms of impact of digital transformation. I think this can clearly show the mechanism of enterprise innovation as a mechanism. In the analysis of the specialization and division of labor and the innovative and important intermediary of specialized production of enterprises, I have explored it from the perspective of enterprise expansion and development. I am aware of the importance of clarifying the law of firm development for the study of this paper. Therefore, by reviewing the literature, I found that the horizontal expansion of enterprises inevitably promotes the efficient use of resources. Moreover, digital transformation can expand the production boundaries of enterprises and enhance specialized production capacity by reducing production costs and management costs. These factors can improve the efficiency of resource utilization, which in turn has a positive effect and impact on capacity utilization. Therefore, the specialized production segmentation of enterprises

Powered by Editorial Manager® and ProduXion Manager® from Aries Systems Corporation

is presented in this section as one of the highlights of this study.
With these in-depth explorations and studies, the first hypothesis – the positive impact of digital transformation on enterprises – is presented here in this study. The theoretical framework is reconstructed to make the logic of the paper seem more reasonable and to make the theory of the paper more accessible and acceptable. (See I. Introduction and II. Theoretical analysis in the revised version for more details)

3.Three hypotheses are not well organized. As far as I'm concerned, H1, H2 and H3 are about a same thing: how digitalization promotes capacity utilization, so the main topic can be summarized into one hypothesis, which can be treated as the paper's main proposition and contribution. As stated in point 2, the authors can write a theoretical model to see whether the U-shaped nonlinear relationship can be derived from the model.
The author's answer: We would like to thank the reviewers for their very professional and pertinent suggestions. Following the suggestions of the reviewers, after further deliberation and careful consideration, I have made the following changes to the hypothesis section of the paper – Hypothesis 1 and Hypothesis 3 are combined, as Hypothesis 2 is retained as a research innovation of this paper.
The specific reasons are as follows: While these assumptions are all about the impact of digital transformation on capacity utilization, there are positive and negative mechanisms of action. A careful study of the literature reveals that the mechanism of action of digital transformation on capacity utilization has not only mediated the positive effects of innovation and specialized production, but also some negative adverse effects may exist. There is no guarantee that digital transformation will necessarily lead to efficient resource utilization. In the case of the communications industry, for example, the overuse of digital services has a more significant negative impact on data-intensive manufacturing. The lack of a better institutional design for advancing digital transformation in the initial stages of digital transformation will lead to a failure to truly improve the efficiency of resource utilization in the enterprise. However, contemporary research has paid little attention to the negative impact of digital transformation on business development, that is, almost all research has focused on the positive impact of digital transformation on business. Therefore, the U-shaped character of the impact of digital transformation on the capacity utilization of firms can be used as an innovative analysis for the paper's research.
I strongly endorse the professional suggestions of the reviewers. Indeed, at the beginning of this research, I also considered whether to construct a theoretical model to explain this mechanism. By referring to a large body of literature and comparing analyses, I found that constructing a theoretical model does not explain the role of digital transformation as well as a theoretical analysis does, nor does it visually demonstrate the impact of digitalization in the enterprise. In general, the mechanism of digital transformation can be more generally understood and described by theoretical analysis, and therefore, after repeated consideration, I have chosen theoretical analysis to describe this mechanism of influence.

Minor comments and typos
1. The authors cite so many Chinese journals, and there are no more than 8 articles cited from international authoritative journals.
The author's answer: This has been increased to 28 articles.

2. The citing format in the article is wrong.
The author's answer: Thank you for the kind corrections. They have been corrected.

3. The formula is not fully displayed, although I can guess what it is.
The author's answer: I am very sorry for this error, which was due to my oversight. I thank the reviewers for their corrections and have made adjustments to confirm that it is fully displayed.

Thank you for giving us the opportunity to submit a revised draft of this manuscript for publication in Plos One. We appreciate the time and effort that you and the reviewers have devoted to providing feedback on our manuscript and are grateful for insightful comments and valuable improvements to our manuscript. We have incorporated most of the suggestions made by the reviewers. These changes are highlighted in the manuscript.
According to the reviewer's comments, we have revised the manuscript extensively. If

	there are any other modifications we could make, we would like very much to modify them and we appreciate your help. We hope that our manuscript will be considered for publication in your journal. Thank you so much for your help.<br><br>Yours sincerely,<br>Ning Zhao<br>November 20, 2022<br>Zhongnan University of Economics and Law
**Additional Information:**	
**Question**	**Response**
**Financial Disclosure**<br><br>Enter a financial disclosure statement that describes the sources of funding for the work included in this submission. Review the submission guidelines for detailed requirements. View published research articles from *PLOS ONE* for specific examples.<br><br>This statement is required for submission and **will appear in the published article** if the submission is accepted. Please make sure it is accurate.<br><br>**Unfunded studies**<br>Enter: *The author(s) received no specific funding for this work.*<br><br>**Funded studies**<br>Enter a statement with the following details:<br>• Initials of the authors who received each award<br>• Grant numbers awarded to each author<br>• The full name of each funder<br>• URL of each funder website<br>• Did the sponsors or funders play any role in the study design, data collection and analysis, decision to publish, or preparation of the manuscript?<br>• **NO** - Include this sentence at the end of your statement: *The funders had no role in study design, data collection and analysis, decision to publish, or preparation of the manuscript.*<br>• **YES** - Specify the role(s) played.<br><br>* typeset	Unfunded studies<br>The author(s) received no specific funding for this work.
**Competing Interests**<br><br>Use the instructions below to enter a competing interest statement for this	NO authors have competing interests<br>The authors have declared that no competing interests exist.

Powered by Editorial Manager® and ProduXion Manager® from Aries Systems Corporation

submission. On behalf of all authors, disclose any competing interests that could be perceived to bias this work—acknowledging all financial support and any other relevant financial or non-financial competing interests.<br><br>This statement is required for submission and **will appear in the published article** if the submission is accepted. Please make sure it is accurate and that any funding sources listed in your Funding Information later in the submission form are also declared in your Financial Disclosure statement.<br><br>View published research articles from *PLOS ONE* for specific examples.<br><br>**NO authors have competing interests**<br><br>Enter: *The authors have declared that no competing interests exist.*<br><br>**Authors with competing interests**<br><br>Enter competing interest details beginning with this statement:<br><br>*I have read the journal's policy and the authors of this manuscript have the following competing interests: [insert competing interests here]*<br><br>* typeset	
**Ethics Statement**<br><br>Enter an ethics statement for this submission. This statement is required if the study involved:<br><br>• Human participants<br>• Human specimens or tissue<br>• Vertebrate animals or cephalopods<br>• Vertebrate embryos or tissues<br>• Field research<br><br>Write "N/A" if the submission does not	N/A

require an ethics statement.<br><br>General guidance is provided below. Consult the submission guidelines for detailed instructions. **Make sure that all information entered here is included in the Methods section of the manuscript.**<br><br>**Format for specific study types**<br><br>**Human Subject Research (involving human participants and/or tissue)**<br>• Give the name of the institutional review board or ethics committee that approved the study<br>• Include the approval number and/or a statement indicating approval of this research<br>• Indicate the form of consent obtained (written/oral) or the reason that consent was not obtained (e.g. the data were analyzed anonymously)<br><br>**Animal Research (involving vertebrate animals, embryos or tissues)**<br>• Provide the name of the Institutional Animal Care and Use Committee (IACUC) or other relevant ethics board that reviewed the study protocol, and indicate whether they approved this research or granted a formal waiver of ethical approval<br>• Include an approval number if one was obtained<br>• If the study involved *non-human primates*, add *additional details* about animal welfare and steps taken to ameliorate suffering<br>• If anesthesia, euthanasia, or any kind of animal sacrifice is part of the study, include briefly which substances and/or methods were applied<br><br>**Field Research**<br><br>Include the following details if this study involves the collection of plant, animal, or other materials from a natural setting:<br>• Field permit number<br>• Name of the institution or relevant body that granted permission	
**Data Availability**	Yes - all data are fully available without restriction

Authors are required to make all data underlying the findings described fully available, without restriction, and from the time of publication. PLOS allows rare exceptions to address legal and ethical concerns. See the PLOS Data Policy and FAQ for detailed information.<br><br>A Data Availability Statement describing where the data can be found is required at submission. Your answers to this question constitute the Data Availability Statement and **will be published in the article**, if accepted.<br><br>**Important:** Stating 'data available on request from the author' is not sufficient. If your data are only available upon request, select 'No' for the first question and explain your exceptional situation in the text box.<br><br>Do the authors confirm that all data underlying the findings described in their manuscript are fully available without restriction?	
**Describe where the data may be found in full sentences. If you are copying our sample text, replace any instances of XXX with the appropriate details.**<br><br>• If the data are **held or will be held in a public repository**, include URLs, accession numbers or DOIs. If this information will only be available after acceptance, indicate this by ticking the box below. For example: *All XXX files are available from the XXX database (accession number(s) XXX, XXX.*).<br>• If the data are all contained **within the manuscript and/or Supporting Information files**, enter the following: *All relevant data are within the manuscript and its Supporting Information files.*<br>• If neither of these applies but you are able to provide **details of access elsewhere**, with or without limitations, please do so. For example:	All relevant data are within the manuscript and its Supporting Information files.

Data cannot be shared publicly because of [XXX]. Data are available from the XXX Institutional Data Access / Ethics Committee (contact via XXX) for researchers who meet the criteria for access to confidential data.<br><br>*The data underlying the results presented in the study are available from (include the name of the third party and contact information or URL).*<br>• This text is appropriate if the data are owned by a third party and authors do not have permission to share the data.<br><br>* typeset	
Additional data availability information:	

Powered by Editorial Manager® and ProduXion Manager® from Aries Systems Corporation

Impact of enterprise digital transformation on capacity utilization: Evidence from China

Ning Zhao[1],Jianxin Ren[1*]

[1] School of Business Administration, Zhongnan University of Economics and Law, No.182, Nanhu Avenue,430073, China

*Corresponding author
E-mail: 202001080002@stu.zuel.edu.cn (J.R.);
Tel.: +86-13277903806

Abstract

This study analyzes data from Chinese listed companies from 2010 to 2018, and demonstrates that enterprises' digital transformation has a significant impact on increasing the rate of capacity utilization,and there is an inverted U-shaped relationship. Digital transformation, in particular, is a significant driving force of enterprise-specific production and innovation. Furthermore, enterprise innovation and enterprise-specialized production play a mediating role in the impact of enterprise digital transformation on capacity utilization. Further analysis of heterogeneity reveals that the impact of digital transformation on capacity utilization is significant for firms with larger capital scales or with poor governance and manufacturing. However, it is less important in enterprises with small and medium capital scales or with more standardized governance, as well as in non-manufacturing (service) enterprises. In summary, this study investigates the mechanisms and economic impact of digital transformation, and its findings have critical theoretical and practical implications.

Keywords: digital transformation; vertical specialization; enterprise innovation; capacity utilization

Introduction

Digital economy is the future direction of global development and a major driving force in the global economy. In comparison to traditional economies, digital economies transcend the limitations of physical economic development factors and

fundamentally alter business organizations' operation logic and value manufacturing methods [1]. As an important focal point for the development of digital economies, enterprise digital transformations use cloud computing, big data, artificial intelligence, and other digital technologies to empower various industries and fields, which have become core drivers to promote quality, efficiency, and power change in economic development and enhance national digital competitiveness. The deep integration of digital technologies and traditional industries is being achieved gradually. Simultaneously, the economic effects of digitalization have gradually gained attention.

Few studies have focused on the impact of information and communication technology on capacity utilization, arguing that IT applications make firms' production more accurate and flexible, reduce information asymmetry between supply and demand, promote firms' technological innovation, reduce external transaction costs, enhance information sharing, and improve production flexibility, which in turn increase the organization's resource utilization efficiency [2,3]. However, a lack of technology, obsolete equipment, unstable power supply, and weak regulations can weaken the effectiveness of information and communication technology [4] and impact capacity utilization.

Several early scholars have explained the factors influencing capacity utilization in terms of market competition. Some researchers [5–8] have contended that the "excessive entry theorem" explains the problem of duplication and overcapacity in oligopolistic markets. Their central claim is that when markets are open to new entrants and Cournot competition is enforced, the number of firms in equilibrium will

be greater than the number that maximizes social welfare. Another stream of literature [9–12] examines excess capacity as an important strategy for firms to compete, and pre-entrants use excess capacity to create credible threats and barriers to entry. Some previous studies have explored the causes of overcapacity from the perspective of the reconfiguration of firms' own operating models and business forms, as well as the combinability of digital technologies, which has resulted in the reconfiguration and nested linking of traditional factors of production [13], the formation of mutually empowering endogenous systems between digital technologies and traditional factors, which have brought profound changes to firms' organizational management and production models [14], and improved the accuracy of corporate investment and production decisions, thereby reshaping the corporate value creation model [15] and necessitating an examination of the changes in corporate capacity utilization from a digital perspective. According to the above theoretical perspective, the majority of enterprises in China, the world’s largest developing country, have a low capacity utilization rate and a more prominent overcapacity, with the local government actively promoting the digital transformation of enterprises. Therefore, this study examines Chinese enterprises as a case study and uses data from Chinese listed companies to investigate the impact of digital transformation on capacity utilization.

When compared to existing related studies, this research makes three important contributions. First, unlike most previous studies that discuss capacity utilization theoretically at the macro and local government levels, this study examines capacity utilization at the micro level. Second, in terms of research framework, this study

analyzes capacity utilization of enterprises by considering the level of enterprise specialized production as an important mediating variable and enterprise heterogeneity, i.e., the corresponding micro characteristics of large enterprises and SMEs for comparative analysis of the impact of digital transformation on their capacity utilization. This is a novel approach as these two factors have never been used to explain capacity utilization in previous studies. Third, in terms of research objective, while most existing studies on the role of digital economies are aimed at reducing resource mismatch and increasing productivity, this study aims to support the proposal that the progress of digital transformation should be combined with actual enterprise development, thereby broadening the research dimension on digital economies.

The remainder of this paper is organized as follows. The following section provides a theoretical analysis of the impact of digital transformation on enterprise capacity and presents the research hypotheses. The section that follows builds an econometric model and explains the construction of indicators and data sources. The empirical analysis, robustness tests, mechanism test, and heterogeneity analysis are presented in the following two sections. Finally, the study’s findings and policy implications are discussed.

Theory and hypotheses

Capacity utilization rate, also known as equipment utilization rate, is the ratio of total industrial output to production equipment; that is, how much of the actual

production capacity is in use. When an enterprise's output is significantly lower than its normal capacity or its product supply significantly exceeds the market demand, the enterprise capacity is idled. Therefore, effective output optimization and a reduction in the degree of resource mismatch are critical for alleviating overcapacity.

Enterprise digital transformation improves technological innovation capabilities, optimizes production and operational processes, and reduces internal and external communication costs [3], providing a feasible path to alleviate corporate overcapacity. In terms of innovation models, digital technology enables the timely interaction between internal and external innovation agents, and cutting-edge technologies can capture a large amount of consumer behavior data to guide corporate innovation [16]. Enterprises can also improve information exchange and collection with inaccessible external organizations through the Internet, allowing them to obtain more resources for more effective innovation strategies [17,18]. Firms with sound big data processing and analysis capabilities can improve organizational learning and help them innovate and develop new products [19]. According to Porter and Heppelmann [20], the arrival of the digital technology revolution has triggered more innovations that improve product performance and functionality, thereby enhancing organizations' operational performance. Consequently, efficient production increases competitive advantage, breaks the product homogeneity dilemma, generates new consumer demand, captures more market share, and increases firm capacity utilization.

Yuan et al. [21] discovered that digitalization promotes firm specialization by lowering their external transaction costs, and that the effect of digitalization on firm

specialization is stronger in firms with low internal control costs, as well as firms in manufacturing and competitive industries. Furthermore, they discovered that digitalization increases total factor productivity by influencing firm boundaries, which has a positive impact on firm economic efficiency. Labor specialization increases productivity, drives technological innovation, and promotes firms' economic growth [22], which improves resource utilization and reduces the level of overcapacity. This is because digital transformation a greater impact on reducing external transaction costs than it does on reducing internal control costs; in other words, the role of driving the movement of corporate boundaries toward a specialized division of labor is dominant. Furthermore, firm production shifts resources from low utilization sectors to high utilization ones, and the horizontal expansion of firms boosts capacity utilization. Thus, the following hypothesis is proposed:

H1: Enterprise digital transformation increases the level of specialization in production and innovation to increase capacity utilization; That is, specialization and product innovation act as mediators in the influence path.

While several studies have examined the impact of digital transformation on enterprises, only a few of them have focused on digital transformation and capacity utilization, particularly the negative impact of digital transformation on business development. Corporate changes are often accompanied by significant uncertainty [23]. Although digital transformation through the advancement of digital technology and infrastructure has already begun, there is still considerable uncertainty about how it will be integrated into existing corporate organizations. Digital transformation, as a

type of change, is not always linear in its progression. Excessive emphasis on digital transformation while overlooking its integration with existing production models and strategic options may be counterproductive to improving capacity utilization due to the following reasons. First, the integration of digital technology and industrial systems is still in its early stages, and “exploratory innovation” as a source of new technology is highly risky. Therefore, if digital transformation is over-promoted, it will not only consume firms’ existing production resources but will also create resource mismatches. Marel et al. [24] attempted to develop regulatory indicators for digital services and discovered that the use of enhanced digital services had a more significant negative impact on data-intensive manufacturing. In the early stages of digital transformation, a lack of a better institutional design to promote the process will result in a failure to truly improve the efficiency of enterprise resource utilization, and the transformation will essentially devolve into optimizing the IT base rather than overall synergy, impeding capacity utilization improvement. According to Peng [25], the negative impact of digital technology on energy consumption includes the indirect effects on economic growth caused by the energy plundering effect. In the communications industry, for example, digital transformation is accompanied by significant energy consumption in the production, use, and disposal of related products. Steffen [26] contends that digitalization has both inhibitory and growth effects on energy consumption. The inhibitory effect is manifested by digitalization’s ability to reduce the intensity of resource inputs by improving resource utilization efficiency and optimizing industrial structure. The growth effect is the direct increase

in energy consumption such as the production, use, and disposal of digital technologies, and the indirect energy demand caused by the development of a digitally enabled economy. Excessive energy consumption has a negative impact on capacity utilization. Currently, firms' digital transformation processes are hampered by difficult data governance and "data silos." In the strategic planning of some enterprises, digital strategy and business development have "two lines and two skins," while digital transformation and business development have a weak correlation. Therefore, the following hypothesis is proposed:

H2: The effect of digital transformation on capacity utilization is shaped like an inverted U.

Sample selection and data

The rapid expansion of China's digital economy corresponds to the rapid development of digital technology, and the trend of widespread use of digital tools began after 2009. Therefore, this study uses A-share non-financial listed companies from 2010 to 2018 as the initial research sample, and relevant financial and governance data are primarily obtained from Wind and CSMAR databases, which in previous years were mainly sourced from the China City Statistical Yearbook. Company innovation and innovation input-related data were compared with Wind database data to check for gaps, and data from different databases were checked by reviewing company annual reports in cases of inconsistencies in the calibration process.

To ensure the study's quality, the sample was screened in the following ways: (1) excluding companies with special treatment status, (2) excluding companies with abnormal financial status, (3) excluding samples with missing relevant data, and (4) tailing at the 1% level for relevant important variables by year. After the screening process, the sample consisted of 15942 company-annual observations. During data collection, Python was used for text mining and Stata16 was used for data processing.

Key variables

Explained variable

Capacity utilization rate (CU): This is the ratio of actual output to output capacity, which is commonly used as a measurement index to study the level of overcapacity. Among the existing capacity measurement methods, the peak, cost function, production function, and data envelopment analysis (DEA) methods have been adopted by several scholars. The production function method is a metric based on the definition of technical capacity. Technical capacity is the level of capacity at which all capital stock, labor, land, and other factors of production invested in industrial production can be fully utilized for maximum output, assuming preferences and technologies are identified. The annual series of capacity data is estimated in the actual application of the production function method for capacity measurement by setting the production function and extrapolating the annual series of actual output to its "boundary" using the measurement method. The production function method is used in this study to calculate the capacity utilization rate of each industry and its

three main product segments. The following are the reasons for using this method. First, the production function method is based on neoclassical growth theory, which is not based on historical experience and has a strong theoretical foundation, good validity, and reliability in measuring capacity utilization. Second, unlike the peak method, the production function method incorporates the influence of technological progress, capital, and labor on output, as well as the degree of contribution of each production factor to output, which can reflect the changes in and characteristics of economic structure and production activities. Third, the production function method requires only data related to the three indicators of capital, labor, and output for practical application, and the production function method has certain advantages over the cost function method in terms of data availability. Finally, given that different production links in each industry's industrial chain involve different subsectors, the maximum production capacity that each production link can achieve varies. Therefore, the production function approach can eliminate production capacity differences between different enterprises in each production chain, making measurements comparable and estimation results directly applicable to the existing capacity assessment standards.

The Cobb-Douglas (C-D) production function is used in this study to model the production function in its basic form as follows:

$$Y_{i,t} = f\left(K_{i,t}, L_{i,t}\right) = A_i K_{i,t}^{\alpha} L_{i,t}^{\beta} e^{-\mu}, i = 1,2,3, t = 1,2, \dots, T$$

where *i* denotes different links in the chain; *t* is the number of sample years; Y is the value of real output, expressed as annual business revenue; $K_{i,t}$ is the fixed capital stock, expressed as annual average net fixed assets; $L_{i,t}$ is the labor input, which, using the standard followed by several scholars, is represented by the annual number of employees; and *A* denotes the technology level, typically used as a fixed constant. The parameters α and β denote the output elasticities of fixed capital stock and labor input, respectively. Assuming constant returns to scale, we have:

$$0 < \alpha, \beta < 1 \text{ 且} \alpha + \beta = 1$$

The logarithm of the above equation would be:

$$lnY_{i,t} = \alpha lnK_{i,t} + \beta lnL_{i,t} + lnA - \mu$$

Extrapolating the production function to its "boundary," the boundary production function is obtained as:

$$lnY_{i,t}^{*} = \alpha lnK_{i,t} + \beta lnL_{i,t} + lnA$$

where $Y_{i,t}{}^{*}$ denotes the theoretical maximum level of output or potential output. Let lnA = α and E(μ) = ε; substituting these variables in the above equation:

$$lnY_{i,t} = \alpha lnK_{i,t} + \beta lnL_{i,t} + (\alpha - \varepsilon) - (\mu - \varepsilon)$$

Since $E(\mu - \varepsilon) = 0$, estimating the above equation using ordinary least squares (OLS) yields:

$$ln\hat{Y}_{i,t} = \hat{\alpha} lnK_{i,t} + \hat{\beta} lnL_{i,t} + (\alpha - \hat{\varepsilon})$$

The boundary production function generated by changing the constant term in the equation above is called the average production function, and it is further treated as follows:

$$Max\left(lnY_{i,t} - ln\hat{Y}_{i,t}\right) = Max\{lnY_{i,t} - [\hat{\alpha}lnK_{i,t} + \hat{\beta}lnL_{i,t} + (\alpha - \hat{\varepsilon})]\}$$

Thus, the boundary production function deduced from the estimation is:

$$\hat{Y}_{i,t} = e^{\hat{\alpha}}K_{i,t}^{\hat{\alpha}}L_{i,t}^{\hat{\beta}}$$

Capacity utilization is measured as:

$$CU = Y_{i,t}/\hat{Y}_{i,t}$$

The capacity utilization rate estimated in this way has a value of 1. In other words, the capacity utilization rate obtained for each listed company is not an absolute value. The maximum capacity utilization rate of the selected sample during the period 2010–2018 is 100%, while the remaining estimates are relative to the maximum of the selected period. Because the maximum capacity that each production link in the industry chain can achieve varies, this treatment eliminates company differences and makes the estimation results directly applicable to the existing capacity evaluation criteria.

Explanatory variable

Degree of digitalization (DT): Given that a standard metric to measure the level of firms' digital transformation is yet to be developed, this study uses Python to mine digital transformation data from the annual reports of listed companies. According to

a review of existing studies on digital transformation, enterprise digital transformation is measured by capturing the use of information technology such as the Internet by enterprises through text analysis [27], but such indicators are unidimensional and focus only on the application of a specific information technology, making it difficult to obtain a comprehensive picture of enterprise digitalization. This study, however, creates a relatively complete digital lexicon using semantic representations of national digital economy policies, and employs machine learning-based text analysis methods to construct a more comprehensive indicator that reflects the degree of digitalization of Chinese listed enterprises. This digital lexicon is then expanded to a Chinese lexicon to conduct a text analysis on the “Management Discussion and Analysis” (MD&A) section of the listed companies’ annual reports based on machine learning to obtain the frequency of digital-related terms in the annual reports. Given that the length of the MD&A sections of annual reports vary between companies, after extracting the frequency of each keyword in each listed company's annual report, the sum of the frequency of digitalization-related words divided by the length of the MD&A sections of annual reports is used to measure the degree of digitalization (DT) of micro-enterprises. This indicator is multiplied by 100 for notational convenience. The higher the value of the DT indicator, the greater the degree of digitalization.

Mediating variables

Firm innovation (Inn): The data for corporate innovation in this study are primarily derived from the number of invention patent applications in the CSMAR database’s

patent sub-database of listed companies and subsidiaries, which is logarithmic processing to ensure data consistency. Data on corporate innovation and innovation investments are compared to Wind database data to identify gaps, and the data are double-checked by consulting the companies' annual reports in the case of a few inconsistencies in the calibration process. Corporate innovation is measured based on the Chinese patent database due to the following reasons. First, it ensures better data integrity as the CSMAR database matches with the Wind database to form long-term panel data. Second, it ensures greater data reliability as patent data is based on corporate innovation behavior, as opposed to "R&D investment" and "new product output value." Finally, it provides a more detailed classification as it assists in distinguishing between different types of enterprise innovation and refining enterprise innovation behavior based on information from different patent applications and licenses. It is noteworthy that patent data contains additional information, such as application information, authorization, and patent citation information. Given that this study focuses on the impact of digital transformation on innovation behavior, the fluctuation in the number of patent applications should reflect this focus; thus, the number of patent applications for inventions are used to measure innovation behavior.

Degree of production specialization (VSI): Vertical integration and specialization are diametrically opposed concepts in labor studies, with higher vertical integration implying lower specialization and vice versa. Value added to sales (VAS), proposed by Adelman [28] as a proxy measure of vertical integration, has been widely used in

management studies thus far. The basic idea is to estimate the value-added to sales revenue ratio; the higher the ratio, the greater the firm's vertical integration. Following previous studies [29,30], this study uses a modified value-added method to estimate VAS as follows:

$$\mathrm{VAS} = \frac{\text{Value added} - \text{net profit after tax} + \text{normal profit}}{\text{Income from main business} - \text{net profit after tax} + \text{normal profit}}$$

$$= \frac{\text{Value Added } - \text{Net Income After Tax} + \text{Net Assets} \times \text{Average Net Asset Margin}}{\text{Income from main business} - \text{net income after tax} + \text{net assets } \times \text{Average net asset margin}}$$

Value added is expressed as the difference between the firm's sales and purchases, and VAS measures value added as a percentage of sales revenue, with larger values indicating greater vertical integration. Following Zhang [31], the inverse of VAS is defined as the degree of firm specialization (VSI). Therefore, VSI is calculated as:

$$VSI = 1 - VAS$$

A higher VSI value indicates a higher level of enterprise specialization. Following Fan Ziying and Peng Fei [30], observations in which the VSI deviates from the reasonable value domain (0,1) interval are excluded from the analysis to ensure the metric's validity .

Other variables

Many factors contribute to firm overcapacity. In addition to the digital transformation of firms, characteristics such as firm size (*size*), gearing (*lev*), return

on total assets (*roa*), and geographical and industry characteristics, such as the nature of firms (*soe*), can have an impact on a firm's capacity utilization. Therefore, this study controls for these variables. Furthermore, *code*, *industry*, *province*, and *year* denote firm, industry, region, and year fixed effects, respectively. Table 1 summarizes the definitions of all the key variables used in this study.

Table 1. Variable definitions

Variable type	Name	Symbols	Measurements
Explained variable	Capacity utilization rate	*CU*	Actual and potential outputs are calculated using the production method (revenue from main business / maximum expected revenue)
Explanatory variable	Digitalization of enterprises	*DT*	Text analysis of listed companies' annual reports using Python to estimate the degree of digital transformation
Mediating variables	Corporate innovation	*Inn*	Logarithm of the number of invention patent applications by listed companies in the year
	Production specialization	*VSI*	The modified value-added method is used to obtain the firm's value-added as a proportion of its sales revenue, measuring the firm's level of vertical integration (VAS), the inverse (1-VAS) of which indicates the level of specialization
Control variables	Company scale	*size*	ln(Number of employees)
	Gearing ratio	*lev*	(Total liabilities at the end of period / total assets) × 100%
	Total return on assets	*roa*	(Total profit – (income tax rate × total profit)) / (average total liabilities + average owner's equity) × 100%
	Nature of business property	*soe*	If the company is a state-owned enterprise, the value is 1; otherwise, it is 0
	Age of the company	*age*	ln(Year of observation+1 - Year of registration)
Fixed effects	Region	*province*	Province of the company
	Industry	*industry*	Industry categories
	Year	*year*	Current year
	Company	*code*	Stock code of listed companies

Empirical model

Following previous studies [21,31], the impact of digital transformation (inverted "U" mechanism), production specialization, enterprise innovation, and capacity utilization are evaluated using theoretical analysis. To test the study's hypotheses, the quadratic terms of the explanatory variables are incorporated, and the following models are developed:

$$CU_{i,t} = \alpha_0 + \alpha_1 DT_{i,t} + \gamma Controls + \varepsilon_{i,t} \quad (1)$$

$$CU_{i,t} = \alpha_0 + \alpha_1 DT_{i,t} + \alpha_2 DT_{i,t}^2 + \gamma Controls + \varepsilon_{i,t} \quad (2)$$

If the coefficients α_1 and α_2 are significantly positive, it means that the enterprise's digital transformation positively promotes its capacity utilization, and the enterprise's capacity increases in tandem with digital transformation. However, if α_1 is significantly positive and α_2 is significantly negative, it means that the impact of the enterprise's digital transformation on its capacity utilization is inverted U-shaped, thereby supporting hypothesis H2. If α_2 is insignificant, it means that the impact of enterprises' digital transformation on their capacity utilization is linear.

Following Wen and Ye [32], the effects of digital transformation, production specialization, firm innovation, and capacity utilization are tested by using mediating variables. Hypothesis H1 is tested using the following models:

$$VSI_{i,t} = \alpha_0 + \alpha_1 DT_{i,t} + \gamma Controls + \sum Firm + \sum Year + \varepsilon_{i,t} \quad (3)$$

$$CU_{i,t} = \alpha_0 + \alpha_1 DT_{i,t} + \beta VSI_{i,t} + \gamma Controls + \sum Firm + \sum Year + \varepsilon_{i,t} \quad (4)$$

$$Inn_{i,t} = \alpha_0 + \alpha_1 DT_{i,t} + \gamma Controls + \sum Firm + \sum Year + \varepsilon_{i,t} \quad (5)$$

$$CU_{i,t} = \alpha_0 + \alpha_1 DT_{i,t} + \beta Inn_{i,t} + \gamma Controls + \sum Firm + \sum Year + \varepsilon_{i,t} \quad (6)$$

$$CU_{i,t} = \alpha_0 + \alpha_1 DT_{i,t} + \theta VSI_{i,t} + \beta Inn_{i,t} + \gamma Controls + \sum Firm + \sum Year + \varepsilon_{i,t} \quad (7)$$

Results

Descriptive analysis

As shown in Table 2, the mean and standard deviation of capacity utilization level (*CU*) are 1.855 and 5.157, respectively, with a maximum value of 219.8 and a minimum value of 0.02, indicating that the degree of capacity utilization varies significantly between enterprises. *DT* has a mean and standard deviation of 0.229 and 0.353, respectively, with a median, maximum, and minimum values of 0.082, 3.637, and 0, respectively. This also shows that the degree of digitalization varies significantly between enterprises. The mean and variance of enterprise specialization (*VSI*) are 0.549 and 0.212, respectively, with maximum and minimum values of 1 and 0, respectively, indicating the significant differences in the level of specialization of different enterprises. Furthermore, statistical results by industry show that there are considerable differences between industries in terms of digitalization development with the following characteristics. (1) rapid development of digitalization in the

service sector; (2) better integration of manufacturing and digital technology is especially noticeable in technology-intensive industries; (3) agriculture, public utilities, and resource-intensive traditional industries have significantly lower digital transformation than the general industry average. Furthermore, there are considerable differences in capacity utilization across industries, with more capital-intensive industries having lower capacity utilization and technology-intensive industries having higher capacity utilization. This is consistent with the conventional view of overcapacity.

Table 2. Descriptive statistics results

variable	N	mean	p50	Sd	min	max	range
CU	15942	1.855	0.960	5.157	0.0190	219.8	219.8
DT	15942	0.229	0.0820	0.353	0	3.637	3.637
DT^2	15942	0.177	0.00700	0.570	0	13.23	13.23
Inn	10766	2.260	2.197	1.463	0	9.108	9.108
VSI	15942	0.549	0.561	0.212	0	1	1
roa	15942	0.0400	0.0380	0.0750	-1.648	4.837	6.485
size	15942	7.710	7.616	1.324	2.565	13.22	10.66
lev	15942	0.427	0.420	0.211	0.00700	0.995	0.988
age	15942	2.057	2.197	0.894	0	3.367	3.367
soe	15942	0.398	0	0.489	0	1	1

Baseline regression results

Table 3 presents the benchmark regression results, where the first column controls only for fixed effects and the second column includes firm- and region-level control variables. The results show that the *DT* coefficients are all significantly positive at less than 1%, implying that the greater the degree of digitalization, the higher the capacity utilization. In terms of economic significance, the results in the second column show that a 1% increase in a firm's degree of digital transformation

increases its capacity utilization by 31.5%. This suggests that, both economically and statistically, digital transformation has a significant incentive effect on enterprise capacity utilization, and hypothesis H1 is supported by these empirical results. As stated in the previous theoretical analysis, firm capacity utilization is determined by the difference between actual output and potential maximum output, and digital transformation of firms may either increase the potential output, exacerbating the overcapacity situation, or increase the actual output level, thereby enhancing firms' capacity utilization. The results in Table 3 show that digital transformation has a significantly greater effect on actual output than potential output, and that enterprise digital transformation significantly increases capacity utilization level, supporting hypothesis H1 in the theoretical model. To verify the validity of hypothesis H2, a quadratic treatment on the explanatory variables is performed to obtain the variable *DT*2. The findings in Table 3's third column show that the *DT*2 coefficient is significantly negative, and when combined with the coefficient of the primary term, it becomes evident that the impact of digital transformation on capacity utilization is nonlinear and inverted U-shaped.

Table 3. Benchmark regression results

Variables	(1)<br>CU	(2)<br>CU	(3)<br>CU
DT	0.315**	0.437**	0.93***
	(0.137)	(0.18)	(0.338)
DT^2			-0.283*
			(0.164)
size2		-1.053***	-1.054***
		(0.065)	(0.065)
roa		1.936***	1.923***
		(0.432)	(0.432)

lev		2.693***	2.703***
		(0.311)	(0.311)
age		-0.34***	-0.345***
		(0.106)	(0.106)
soe		-0.141	-0.138
		(0.24)	(0.24)
Constants	1.727***	9.406***	9.359***
	(0.087)	(0.516)	(0.517)
Observations	15942	15912	15912
Adj-R^2	0.673	0.673	0.673

Note: t-statistics adjusted for firm-level clustering are in parentheses; ***, **, and * indicate significance at the 1%, 5%, and 10% levels, respectively, and the same as in the following tables, if not otherwise specified.

Robustness tests

Endogenous problems

Previous studies may have raised endogeneity concerns for the following two reasons: (a) increased digital transformation of enterprises will improve their production and business environment, as well as promote higher levels of capacity utilization; (b) increased capacity utilization may also necessitate high-level digital development of enterprises, weakening unnecessary trade resistance and assisting them in improving production efficiency. The instrumental variables approach is used in this study to mitigate the reverse causality of the aforementioned potential endogenous effect on the findings.

Following Huang et al. [34], the instrumental variables for firm digitalization in this study are based on 1984 postal data for each city. This is done primarily because the communication methods used in the previous development process in the firm's location will influence the firm's application and acceptance of information technology in terms of technology level and social preferences during the sample

period, satisfying the correlation condition. Postal and telecommunications, as social infrastructure, primarily provide communication services to the population and do not directly influence firms' capacity utilization processes, thereby satisfying the exogeneity condition. Furthermore, because the 1984 post and telecommunications information for each city is cross-sectional data that is difficult to use directly as an instrumental variable for panel data, this study follows Yuan Chun et al. [21] and uses the cross-product term of the number of national Internet accesses with a lag of one period and the number of fixed telephones per 10,000 people at the end of 1984 as an instrumental variable for the digitalization progress of enterprises in the current period. The results in column (1) of Table 4 show that the explanatory variable coefficients are significantly positive at the 5% level, thereby demonstrating the validity of the study's primary findings.

Alternative measures of digital transformation

Several methods are employed in this study to arrive at alternative measures of a company's digital transformation. The quantification of digital transformation in the sample firms is based on the frequency of special words of a digital nature appearing in the firms' annual statements, which reflects the importance of the firms' digital transformation. The quantification, however, does not consider firms' investment in the development of digital infrastructure. Therefore, by using digital technology applications and digital technology devices as proxy variables for enterprise digital transformation in the robustness test, this study strengthens the examination of the

conditions under which digital transformation can be achieved. Specifically, they represent the number of people employed in information transmission, computer services, and the software industry (10 million) and the number of Internet broadband access subscribers (100 million) in the year in the city where the listed company is located. According to the results shown in columns (2) and (3) of Table 4, the explanatory variable coefficients are significantly positive, indicating that the results of this study remain robust. Particularly, the degree of digital transformation, which has been replaced by the number of Internet broadband access subscribers, has a significant coefficient at the 1% level, indicating that the direction and significance of the regression coefficients remain the same as previously stated, thereby evidencing the robustness of the primary findings.

Robustness tests

Excess capacity (*OC*), the inverse variable of capacity utilization, is used to replace the explanatory variable as an alternative measure. Column (4) of Table 4 shows that the explanatory variable coefficient is significantly negative, and because the alternative measure is an inverse variable of capacity utilization, this indicates that digitalization significantly suppresses corporate overcapacity and supports the robustness of the study’s primary findings. Regional and industry fixed effects are included in column (5), which are the interaction fixed effects of province and year, and industry and year of listed companies. The explanatory variable coefficients are significant at the 5% level, thereby proving the robustness of the primary findings.

Table 4. Results of robustness tests

Variables	(1) CU	(2) CU	(3) CU	(4) OC	(5) CU
DT_w1	4.567**			-0.099*	0.469**
	(2.106)			(0.055)	(0.198)
news		0.009*			
		(0.005)			
Int us			10.526***		
			(3.068)		
size2	-0.596***	-0.997***	-0.992***	0.865***	-1.043***
	(0.038)	(0.071)	(0.071)	(0.024)	(0.071)
roa	3.818***	1.668***	1.66***	-1.183***	1.785***
	(0.603)	(0.444)	(0.444)	(0.109)	(0.444)
lev	4.818***	2.612***	2.641***	-0.15	2.707***
	(0.275)	(0.33)	(0.329)	(0.095)	(0.329)
age	0.258***	-0.341***	-0.342***	0.113***	-0.334***
	(0.067)	(0.118)	(0.118)	(0.032)	(0.117)
soe	0.195	-0.114	-0.106	-0.093	-0.277
	(0.135)	(0.265)	(0.265)	(0.075)	(0.255)
Constants	2.614***	9.015***	8.743***	13.455***	9.363***
	(0.47)	(0.568)	(0.575)	(0.176)	(0.565)
Observations	15087	14751	14751	8161	15909
Adj-R^2	0.061	0.69	0.691	0.802	0.686

Additional Analyses

Mechanism testing and analysis

The analyses thus far reveal that the mechanism of digital transformation to promote enterprise capacity utilization is to promote enterprise innovation and production specialization, which, in turn, increases the efficiency of enterprises' resource utilization. Therefore, the degree of production specialization (*VSI*) and technological innovation are included in the analysis and hypothesis H1 is proposed, the regression results of which are shown in Table 5.

Table 5. Heterogeneity analysis results

	(1) VSI	(2) CU	(3) Inn	(4) CU	(5) CU
DT	0.023***	0.399**	0.209***	0.102	0.088
	(0.007)	(0.18)	(0.05)	(0.064)	(0.064)
VSI		1.633***			0.71***
		(0.221)			(0.087)
Inn				0.07***	0.069***
				(0.014)	(0.014)
size	0.002	-1.056***	0.321***	-0.857***	-0.851***
	(0.003)	(0.065)	(0.021)	(0.028)	(0.028)
roa	0.145***	1.698***	0.434**	4.53***	4.316***
	(0.017)	(0.432)	(0.212)	(0.273)	(0.273)
lev	0.122***	2.494***	0.223**	1.735***	1.612***
	(0.012)	(0.311)	(0.096)	(0.123)	(0.124)
age	0.002	-0.343***	0.021	-0.008	-0.015
	(0.004)	(0.106)	(0.031)	(0.04)	(0.04)
soe	-0.018**	-0.111	-0.077	-0.121	-0.1
	(0.009)	(0.24)	(0.067)	(0.086)	(0.086)
Constants	0.473***	8.633***	-.406**	7.115***	6.752***
	(0.02)	(0.526)	(0.166)	(0.214)	(0.217)
Observations	15912	15912	10589	10589	10589
Adj-R^2	0.708	0.674	0.806	0.855	0.856

The results in column (1) of Table 5 show that enterprise digital transformations are significantly and positively related to production specialization at the 1% level, which can be interpreted as moderate digital transformation assisting enterprises to exploit their comparative advantages in resources and technology, improve their transaction environment, reduce their internal control costs, and promote their specialization. Column (2) demonstrates that the *VSI* coefficient is significantly positive at the 1% level, whereas the *DT* coefficient decreases in both value and significance. Thus, the combined effect test using the stepwise regression method confirms that digital transformation improves capacity utilization by encouraging firms to achieve production specialization. Column (3) shows that at the

1% level, firm digital transformation significantly promotes technological innovation, probably because digital transformation stimulates product and process innovation, which in turn promotes firms' technological innovation. Column (4) shows that the coefficient of firms' technological innovation is significantly positive at the 1% level, but the coefficient of digital transformation decreases in value and is not significant at all, indicating that the effect of *DT* on firms' capacity utilization is translated into technological innovation, and thus there is technological innovation promoting the capacity utilization of firms. Such technological innovation has a well-mediated impact. Column (5) incorporates all mediating variables into the model, and the comparison reveals that the *VSI* coefficient is significantly larger than the coefficient of technological innovation for the two mediating variables that are also significant at the 1% level. This indicates that both increased specialized production capacity and technological innovation can positively influence firms' capacity utilization, but the effect of specialized production capacity is stronger. However, when the explanatory variable coefficients (*DT*) are compared, the effect of digital transformation is not significant, despite having a positive impact on capacity utilization.

Heterogeneity tests

As discussed in the previous theoretical analysis, digitalization alters the internal and external environments of enterprises, affecting their capacity utilization level. Specifically, digitalization improves enterprise capacity utilization by promoting innovation and production specialization. However, the levels of enterprise capacity utilization and digital transformation vary across enterprises and industries,

which may result in heterogeneity in the effect of digital transformation on enterprise capacity utilization. Therefore, this study's sample is further subdivided to examine the performance of heterogeneity based on corporate governance, company size, and industry dimensions.

Heterogeneity analysis based on enterprise size

Columns (1) and (2) of Table 6 present the estimation results for two subsamples: firms with less than average capital size and firms with greater than average capital size. The estimated coefficient of *DT* in the sample of firms with smaller capital size is significant at the 1% level. However, the regression results for the sample of firms with larger capital are not significant, and the coefficients are negative. This suggests that the digital transformation of firms with smaller capital can effectively contribute to an increase in their capacity utilization by stimulating firm innovation and enhancing production specialization. However, in firms with larger capital, digital transformation has no significant effect on capacity utilization. There may be more resistance to alleviating overcapacity in such enterprises, which reduces the impact of digital transformation. Therefore, larger capital-intensive firms find it challenging to improve their capacity utilization through digital transformation.

Heterogeneity analysis based on corporate governance level

Columns (3) and (4) of Table 6 present the subsample regression results for firms with two different levels of governance. The estimated *DT* coefficient in the sample with poorer governance is significantly positive at the 1% level. Although

numerically positive, the regression results for the sample with higher levels of governance are not significant. This suggests that firms with lower levels of governance will benefit from digital transformation by stimulating innovation and improving specialized production, which will lead to an increase in capacity utilization. Digital transformation can compensate for deficits in capacity utilization caused by a lack of corporate governance in poorly governed firms by improving their resource allocation capabilities. Conversely, the impact of digital transformation on capacity utilization is not significant in firms with a sound governance system. This could be because companies with higher governance levels have greater resource allocation capabilities, making the effect of further improvement in capacity utilization levels due to digital transformation less pronounced. Therefore, corporate governance can be regarded as a substitute for digital transformation.

Heterogeneity analysis based on industry

Columns (5) and (6) of Table 6 present the regression results for manufacturing and service firms, respectively. The estimated *DT* coefficient for the sample of manufacturing firms is significantly positive at the 5% level. The regression results for the sample of service firms, however, are not significant, despite being positive. This suggests that, for manufacturing firms, the digital transformation can contribute to increased capacity utilization through corporate innovation and improved production specialization. In contrast, the role of digital transformation in improving overcapacity of service firms is not significant. The most likely reason for this is that manufacturing companies have significant over-capacities and potential to improve

capacity utilization. Service firms, however, have lower transaction information friction and higher capacity utilization, making the impact of digital transformation less pronounced.

Table 6. Regression results of mechanism analysis

	(1)	(2)	(3)	(4)	(5)	(6)
	CU	CU	CU	CU	CU	CU
DT	-0.364	0.219***	0.187	0.484**	0.151**	0.175
	(0.312)	(0.047)	(0.308)	(0.217)	(0.075)	(0.438)
size	-2.238***	-.473***	-1.312***	-.914***	-.711***	-1.913***
	(0.104)	(0.022)	(0.101)	(0.091)	(0.025)	(0.169)
roa	2.225**	0.539***	3.903***	1.194***	1.355***	3.843***
	(1.074)	(.09)	(.965)	(.455)	(.137)	(1.489)
lev	1.949***	.835***	2.58***	2.324***	1.223***	5.608***
	(0.529)	(0.087)	(0.491)	(0.407)	(0.108)	(0.896)
age	-0.229	0.062**	-0.227	-0.506***	-0.012	-0.257
	(0.217)	(0.029)	(0.208)	(0.138)	(0.036)	(0.313)
soe	-0.063	-0.162**	-0.095	-0.143	-0.092	-0.105
	(0.323)	(0.079)	(0.292)	(0.486)	(0.079)	(0.694)
Constants	21.146***	3.943***	11.985***	7.992***	6.192***	15.614***
	(1.024)	(0.146)	(0.918)	(0.667)	(0.193)	(1.344)
Observations	7844	7830	8125	7411	8629	4234
Adj-R^2	0.844	0.856	0.765	0.591	0.712	0.819

Discussion

Overcapacity has resulted in significant resource misallocation, and waste remains a major issue for economic growth that must be addressed. Most previous studies on the path and methods to enhance capacity utilization have been conducted from a macro perspective, such as government control, business environment, state-owned enterprise soft budgets, and market-oriented mechanisms, seldom investigating the role of digital transformation at an enterprise level. The deep integration of real economies and digitalization has become a trend and an inevitable

result of future development since the advent of the digital era. The healthy development of the digital economy promotes the construction of a modern economic system. As a new type of production factor, data has a significant impact on changes in traditional production methods. The digital economy is not only a new economic growth point, but also a fulcrum for transforming and upgrading traditional industries, and can be an important engine for building a modernized economic system, with high innovation, strong penetration, and widespread coverage. In this regard, no systematic study has been conducted thus far on how digital transformations of enterprises improve overcapacity, and few studies have extensively examined the alternative roles of digital transformation and corporate governance.

Unlike previous studies that investigated capacity utilization governance from macro and regional perspectives, this study examines the impact of digital transformation on capacity utilization from an enterprise-level micro perspective. This study elucidates the theoretical mechanism by which the characteristics of digital transformation affect enterprise innovation and resource allocation efficiency; in other words, how enterprise digital transformation can effectively promote enterprise technological innovation, improve resource allocation efficiency, strengthen the firm’s production specialization, and thus effectively alleviate overcapacity. Furthermore, this study’s findings demonstrate that the effect of digital transformation on capacity utilization is inverted U-shaped, and that relying solely on digital transformation to suppress enterprise overcapacity is ineffective. The impact of digital transformation on capacity utilization is found to be more significant for enterprises

with smaller capital and poorer governance. Moreover, due to the lower likelihood of information mismatch in service firms, digital transformation appears to alleviate overcapacity more effectively in manufacturing enterprises than in service organizations.

It should be pointed out that although this paper uses the text analysis method of machine learning to carry out the overall situation of enterprise digital transformation, it has not yet achieved an accurate depiction of the digital aspects of the enterprise (such as research and development, production, sales, etc.). It is worth further research and exploration in the future to further characterize the digital transformation of each link in the enterprise, to investigate more carefully the impact of the mechanism of each link's digital transformation on the external transaction costs and internal control costs of the enterprise.

Acknowledgements: I want to thank Shi Junwei, Liu Xinglin, Hu Shan and Guo Yanbing. I also want to thank the Center for Industrial Economics.

References

1. Bertani, F., Ponta, L., Raberto, M., Teglio, A., & Cincotti, S. (2021). The complexity of the intangible digital economy: an agent-based model. Journal of business research, 129, 527-540. https://doi.org/10.1016/j.jbusres.2020.03.041

2. Marini, G., & Pannone, A. (2007). Capital and capacity utilization revisited: A theory for ICT-assisted production systems. Structural Change and Economic Dynamics, 18(2), 231-248. https://doi.org/10.1016/j.strueco.2007.01.001

3. Dana Jr, J. D., & Orlov, E. (2014). Internet penetration and capacity utilization in the US airline industry. American Economic Journal: Microeconomics, 6(4), 106-37. https://doi.org/10.1257/mic.6.4.106

4. Teryima, S. J., & Sunday, A. (2015). The role of information communication technology (ICT) in enhancing productivity in local government administration in Benue State, Nigeria. International Journal of Business and

Economic Development (IJBED), 3(1), 110-124

5. Spence, M. (1976). Informational aspects of market structure: An introduction. The Quarterly Journal of Economics, 591-597.
https://doi.org/10.2307/1885323

6. Salop, S. C. (1979). Monopolistic competition with outside goods. The Bell Journal of Economics, 141-156.
https://doi.org/10.2307/3003323

7. von Weizsacker, C. C. (1980). A welfare analysis of barriers to entry. The bell journal of economics, 399-420.
https://doi.org/10.2307/3003371

8. Mathis, S., & Koscianski, J. (1997). Excess capacity as a barrier to entry in the US titanium industry. International Journal of Industrial Organization, 15(2), 263-281.
https://doi.org/10.1016/0167-7187(95)01003-3

9. Spence, A. M. (1977). Entry, capacity, investment and oligopolistic pricing. The Bell Journal of Economics, 534-544.
https://doi.org/10.2307/3003302

10. Ghemawat, P. (1984). Capacity expansion in the titanium dioxide industry. The Journal of Industrial Economics, 145-163.
https://doi.org/10.2307/2098506

11. Kirman, W. I., & Masson, R. T. (1986). Capacity signals and entry deterrence. International journal of industrial organization, 4(1), 25-42.
https://doi.org/10.1016/0167-7187(86)90045-7

12. Mathis, S., & Koscianski, J. (1997). Excess capacity as a barrier to entry in the US titanium industry. International Journal of Industrial Organization, 15(2), 263-281.
https://doi.org/10.1016/0167-7187(95)01003-3

13. Holmström, J. (2018). Recombination in digital innovation: Challenges, opportunities, and the importance of a theoretical framework. Information and organization, 28(2), 107-110.
https://doi.org/10.1016/j.infoandorg.2018.04.002

14. Yoo, Y., Henfridsson, O., & Lyytinen, K. (2010). Research commentary—the new organizing logic of digital innovation: an agenda for information systems research. Information systems research, 21(4), 724-735.

15. Vargo, S. L., & Lusch, R. F. (2016). Institutions and axioms: an extension and update of service-dominant logic. Journal of the Academy of marketing Science, 44(1), 5-23.
https://doi.org/10.1007/s11747-015-0456-3

16. Huang, J., Henfridsson, O., Liu, M. J., & Newell, S. (2017). Growing on steroids: Rapidly scaling the user base of digital ventures through digital innovation. MIS quarterly, 41(1).
10.25300/MISQ/2017/41.1.16

17. Abouzeedan, A., Klofsten, M., & Hedner, T. (2013). Internetization management as a facilitator for managing innovation in high-technology smaller firms. Global Business Review, 14(1), 121-136.
https://doi.org/10.1177/0972150912466462

18. Reim, W., Parida, V., & Örtqvist, D. (2015). Product–Service Systems (PSS) business models and tactics–a systematic literature review. Journal of Cleaner Production, 97, 61-75.
https://doi.org/10.1016/j.jclepro.2014.07.003

19. Tan, K. H., Zhan, Y., Ji, G., Ye, F., & Chang, C. (2015). Harvesting big data to enhance supply chain innovation capabilities: An analytic infrastructure based on deduction graph. International Journal of Production Economics, 165, 223-233.

https://doi.org/10.1016/j.ijpe.2014.12.034

20. Porter, M. E., & Heppelmann, J. E. (2015). How smart, connected products are transforming companies. Harvard business review, 93(10), 96-114.

21. YANG De-ming; LIU Yong-wen. Why Can Internet Plus Increase Performance,[J]China Industrial Economics,2018(05):80-98. https://doi.org/10.19581/j.cnki.ciejournal.2021.09.007

22. Adam Smith. The Wealth of Nations[M]. Chicago: Univexrsity of Chicago Press, 1776

23. Eisenhardt, K. M. (1989). Making fast strategic decisions in high-velocity environments. Academy of Management journal, 32(3), 543-576.

24. Crozet, M., & Milet, E. (2017). Should everybody be in services? The effect of servitization on manufacturing firm performance. Journal of Economics & Management Strategy, 26(4), 820-841. https://doi.org/10.1111/jems.12211

25. Peng, G. C. A. (2013). Green ICT: A strategy for sustainable development of China's electronic information industry. China: An International Journal, 11(3), 68-86.

26. Lange, S., Pohl, J., & Santarius, T. (2020). Digitalization and energy consumption. Does ICT reduce energy demand?. Ecological Economics, 176, 106760. https://doi.org/10.1016/j.ecolecon.2020.106760

27. Yang, Deming, Liu, & Yongwen, 2018 Why Can Internet Plus Increase Performance,[J]China Industrial Economics, (05):80-98. https://doi.org/10.19581/j.cnki.ciejournal.2018.05.005

28. Adelman, M. A. (1955). Concept and statistical measurement of vertical integration. In Business concentration and price policy (pp. 281-330). Princeton University Press.

29. Buzzell, R. D. (1983). Is vertical integration profitable. Harv. Bus. Rev.;(United States), 61(1).

30. Fan Ziying, & Peng Fei, 2017 The Effects of ”Business Tax Replaced with VAT Reform”on Firms’ Tax Cuts and Industrial Division Based on the Perspective of Industrial Interconnection,[J]Economic Research Journal,52(02):82-95. CNKI:SUN:JJYJ.0.2017-02-007

31. Zhang, Y..Vertical Specialization of Firms: Evidence from China's Manufacturing Sector.[R]. CCER Series Paper, 2004.

32. Bing-Zhan Shi & Jian-Tong Li, 2020 Does the Internet Promote Division of Labor? Evidence from Chinese Manufacuring Enterprises,[J].Journal of Management World,36(04):130-149. https://doi.org/10.19744/j.cnki.11-1235/f.2020.0058

34. Wen & Ye, 2014 Analyses of Mediating Effects:The Development of Methods and Models,[J]Advances in Psychological Science,22(05):731-745. 10.3724/SP.J.1042.2014.00731

35. HUANG Qun-hui; YU Yong-ze; ZHANG Song-lin. Internet Development and Productivity Growth in Manufacturing Industry:Internal Mechanism and China Experiences,[J]China Industrial Economics,2019(08):5-23. doi:10.19581/j.cnki.ciejournal.2019.08.001.

collecting data

Click here to access/download
Supporting Information - Compressed/ZIP File Archive
DT date.22.12.07.zip

Revised Manuscript with Track Changes

Impact of enterprise digital transformation on~~and~~ capacity utilization: Evidence from China

Ning Zhao[1],Jianxin Ren[1*]

[1] School of Business Administration, Zhongnan University of Economics and Law, No.182, Nanhu Avenue,430073, China

*Corresponding author
E-mail: 202001080002@stu.zuel.edu.cn (J.R.);
Tel.: +86-13277903806

Formatted: Font: 18 pt

Formatted: Font: 18 pt

Formatted: Font: (Default) Times New Roman Regular, Font color: Auto, (Asian)

Abstract

This study analyzes data from Chinese listed companies from 2010 to 2018, and demonstrates that enterprises' digital transformation has a significant impact on increasing the rate of capacity utilization,and there is an inverted U-shaped relationship. Digital transformation, in particular, is a significant driving force of enterprise-specific production and innovation. Furthermore, enterprise innovation and enterprise-specialized production play a mediating role in the impact of enterprise digital transformation on capacity utilization. Further analysis of heterogeneity reveals that the impact of digital transformation on capacity utilization is significant for firms with larger capital scales or with poor governance and manufacturing. However, it is less important in enterprises with small and medium capital scales or with more standardized governance, as well as in non-manufacturing (service) enterprises. In summary, this study investigates the mechanisms and economic impact of digital transformation, and its findings have critical theoretical and practical implications.

Keywords: digital transformation; vertical specialization; enterprise innovation; capacity utilization

Introduction

Digital economy is the future direction of global development and a major driving force in the global economy. In comparison to traditional economies, digital economies transcend the limitations of physical economic development factors and

fundamentally alter business organizations' operation logic and value manufacturing methods [1]. As an important focal point for the development of digital economies, enterprise digital transformations use cloud computing, big data, artificial intelligence, and other digital technologies to empower various industries and fields, which have become core drivers to promote quality, efficiency, and power change in economic development and enhance national digital competitiveness. The deep integration of digital technologies and traditional industries is being achieved gradually. Simultaneously, the economic effects of digitalization have gradually gained attention.

Few studies have focused on the impact of information and communication technology on capacity utilization, arguing that IT applications make firms' production more accurate and flexible, reduce information asymmetry between supply and demand, promote firms' technological innovation, reduce external transaction costs, enhance information sharing, and improve production flexibility, which in turn increase the organization's resource utilization efficiency [2,3]. However, a lack of technology, obsolete equipment, unstable power supply, and weak regulations can weaken the effectiveness of information and communication technology [4] and impact capacity utilization.

Several early scholars have explained the factors influencing capacity utilization in terms of market competition. Some researchers [5–8] have contended that the "excessive entry theorem" explains the problem of duplication and overcapacity in oligopolistic markets. Their central claim is that when markets are open to new entrants and Cournot competition is enforced, the number of firms in equilibrium will

be greater than the number that maximizes social welfare. Another stream of literature [9–12] examines excess capacity as an important strategy for firms to compete, and pre-entrants use excess capacity to create credible threats and barriers to entry. Some previous studies have explored the causes of overcapacity from the perspective of the reconfiguration of firms' own operating models and business forms, as well as the combinability of digital technologies, which has resulted in the reconfiguration and nested linking of traditional factors of production [13], the formation of mutually empowering endogenous systems between digital technologies and traditional factors, which have brought profound changes to firms' organizational management and production models [14], and improved the accuracy of corporate investment and production decisions, thereby reshaping the corporate value creation model [15] and necessitating an examination of the changes in corporate capacity utilization from a digital perspective. According to the above theoretical perspective, the majority of enterprises in China, the world's largest developing country, have a low capacity utilization rate and a more prominent overcapacity, with the local government actively promoting the digital transformation of enterprises. Therefore, this study examines Chinese enterprises as a case study and uses data from Chinese listed companies to investigate the impact of digital transformation on capacity utilization.

When compared to existing related studies, this research makes three important contributions. First, unlike most previous studies that discuss capacity utilization theoretically at the macro and local government levels, this study examines capacity utilization at the micro level. Second, in terms of research framework, this study

analyzes capacity utilization of enterprises by considering the level of enterprise specialized production as an important mediating variable and enterprise heterogeneity, i.e., the corresponding micro characteristics of large enterprises and SMEs for comparative analysis of the impact of digital transformation on their capacity utilization. This is a novel approach as these two factors have never been used to explain capacity utilization in previous studies. Third, in terms of research objective, while most existing studies on the role of digital economies are aimed at reducing resource mismatch and increasing productivity, this study aims to support the proposal that the progress of digital transformation should be combined with actual enterprise development, thereby broadening the research dimension on digital economies.

The remainder of this paper is organized as follows. The following section provides a theoretical analysis of the impact of digital transformation on enterprise capacity and presents the research hypotheses. The section that follows builds an econometric model and explains the construction of indicators and data sources. The empirical analysis, robustness tests, mechanism test, and heterogeneity analysis are presented in the following two sections. Finally, the study's findings and policy implications are discussed.

Theory and hypotheses

Capacity utilization rate, also known as equipment utilization rate, is the ratio of total industrial output to production equipment; that is, how much of the actual

production capacity is in use. When an enterprise's output is significantly lower than its normal capacity or its product supply significantly exceeds the market demand, the enterprise capacity is idled. Therefore, effective output optimization and a reduction in the degree of resource mismatch are critical for alleviating overcapacity.

Enterprise digital transformation improves technological innovation capabilities, optimizes production and operational processes, and reduces internal and external communication costs [3], providing a feasible path to alleviate corporate overcapacity. In terms of innovation models, digital technology enables the timely interaction between internal and external innovation agents, and cutting-edge technologies can capture a large amount of consumer behavior data to guide corporate innovation [16]. Enterprises can also improve information exchange and collection with inaccessible external organizations through the Internet, allowing them to obtain more resources for more effective innovation strategies [17,18]. Firms with sound big data processing and analysis capabilities can improve organizational learning and help them innovate and develop new products [19]. According to Porter and Heppelmann [20], the arrival of the digital technology revolution has triggered more innovations that improve product performance and functionality, thereby enhancing organizations' operational performance. Consequently, efficient production increases competitive advantage, breaks the product homogeneity dilemma, generates new consumer demand, captures more market share, and increases firm capacity utilization.

Yuan et al. [21] discovered that digitalization promotes firm specialization by lowering their external transaction costs, and that the effect of digitalization on firm

specialization is stronger in firms with low internal control costs, as well as firms in manufacturing and competitive industries. Furthermore, they discovered that digitalization increases total factor productivity by influencing firm boundaries, which has a positive impact on firm economic efficiency. Labor specialization increases productivity, drives technological innovation, and promotes firms' economic growth [22], which improves resource utilization and reduces the level of overcapacity. This is because digital transformation a greater impact on reducing external transaction costs than it does on reducing internal control costs; in other words, the role of driving the movement of corporate boundaries toward a specialized division of labor is dominant. Furthermore, firm production shifts resources from low utilization sectors to high utilization ones, and the horizontal expansion of firms boosts capacity utilization. Thus, the following hypothesis is proposed:

H1: Enterprise digital transformation increases the level of specialization in production and innovation to increase capacity utilization; That is, specialization and product innovation act as mediators in the influence path.

While several studies have examined the impact of digital transformation on enterprises, only a few of them have focused on digital transformation and capacity utilization, particularly the negative impact of digital transformation on business development. Corporate changes are often accompanied by significant uncertainty [23]. Although digital transformation through the advancement of digital technology and infrastructure has already begun, there is still considerable uncertainty about how it will be integrated into existing corporate organizations. Digital transformation, as a

type of change, is not always linear in its progression. Excessive emphasis on digital transformation while overlooking its integration with existing production models and strategic options may be counterproductive to improving capacity utilization due to the following reasons. First, the integration of digital technology and industrial systems is still in its early stages, and "exploratory innovation" as a source of new technology is highly risky. Therefore, if digital transformation is over-promoted, it will not only consume firms' existing production resources but will also create resource mismatches. Marel et al. [24] attempted to develop regulatory indicators for digital services and discovered that the use of enhanced digital services had a more significant negative impact on data-intensive manufacturing. In the early stages of digital transformation, a lack of a better institutional design to promote the process will result in a failure to truly improve the efficiency of enterprise resource utilization, and the transformation will essentially devolve into optimizing the IT base rather than overall synergy, impeding capacity utilization improvement. According to Peng [25], the negative impact of digital technology on energy consumption includes the indirect effects on economic growth caused by the energy plundering effect. In the communications industry, for example, digital transformation is accompanied by significant energy consumption in the production, use, and disposal of related products. Steffen [26] contends that digitalization has both inhibitory and growth effects on energy consumption. The inhibitory effect is manifested by digitalization's ability to reduce the intensity of resource inputs by improving resource utilization efficiency and optimizing industrial structure. The growth effect is the direct increase

in energy consumption such as the production, use, and disposal of digital technologies, and the indirect energy demand caused by the development of a digitally enabled economy. Excessive energy consumption has a negative impact on capacity utilization. Currently, firms' digital transformation processes are hampered by difficult data governance and "data silos." In the strategic planning of some enterprises, digital strategy and business development have "two lines and two skins," while digital transformation and business development have a weak correlation. Therefore, the following hypothesis is proposed:

H2: The effect of digital transformation on capacity utilization is shaped like an inverted U.

~~III. Econometric model and variable setting~~

~~1.~~ Sample selection and data ~~sources~~

The rapid expansion of China's digital economy corresponds to ~~scales with~~ the rapid development of digital technology, and the trend of widespread use of digital tools began, ~~was is mainly reflected~~ after 2009. Therefore, this study~~paper~~ uses~~takes~~ A-share non-financial listed companies from 2010 to 2018 as the initial research sample, and relevant financial and governance data are primarily~~mainly~~ obtained from Wind and CSMAR databases, which in previous years were~~and regional-level data are~~ mainly sourced from~~due to~~ the China City Statistical Yearbook~~ in previous years~~. Company innovation and innovation input-related data were compared with W~~W~~ind database data to check for gaps, and data from different databases were checked by

Formatted: Font: (Default) Times New Roman, 18 pt

Formatted: Line spacing: Double

Formatted: Font: (Default) Times New Roman, 16 pt, Bold

Formatted: Font: (Default) Times New Roman, 12 pt

Formatted: Indent: First line: 2.7 ch, Line spacing: Double

reviewing company annual reports in ~~the ~~cases of~~ a few~~ inconsistencies in the calibration process.

To ensure the study’s quality~~ of the study~~, the sample was screened in the following ways~~ as follows~~: (1) excluding~~eliminating~~ companies with~~in~~ special treatment status,~~;~~ (2) excluding~~eliminating~~ companies with abnormal financial status,~~;~~ (3) excluding samples~~eliminating the sample~~ with ~~serious ~~missing relevant data,~~;~~ and (4) ~~doing ~~tailing at the 1% level for relevant important variables by year. After the screening process, the sample consisted of~~Finally, we obtained data containing~~ 15942 company-annual observations. During data collection, Python was used for text mining~~ during data collection,~~ and Stata16 was used for data processing~~ was performed using~~~~with Stata16~~.

~~2. ~~Key variables~~ metrics~~

~~(1) ~~Explained variable~~:~~

Capacity utilization rate (CU): This is the ratio of actual output to output capacity, which is commonly used as a measurement index to study the level of overcapacity.~~.~~ A~~t present, a~~mong the existing capacity measurement methods, the peak~~ method~~, cost function~~ method~~, production function~~ method~~, and data envelopment analysis (DEA) methods have been~~are~~ adopted by several~~many~~ scholars.~~ When scholars at home and abroad study the level of overcapacity, they usually use the capacity utilization rate (the ratio of actual output to capacity output) as a~~~~the measurement index.~~

Formatted: Font: (Default) Times New Roman

Formatted: Line spacing: Double

Formatted: Font: (Default) Times New Roman, 16 pt

Formatted: Font: (Default) Times New Roman, 16 pt, Bold

Formatted: Font: (Default) Times New Roman, 16 pt

Formatted: Font: (Default) Times New Roman, 16 pt, Bold

Formatted: Font: (Default) Times New Roman, 16 pt

Formatted: Font: (Default) Times New Roman, 14 pt, Bold

Formatted: Font: (Default) Times New Roman, 14 pt

Formatted: Font: (Default) Times New Roman, 12 pt, Italic

Formatted: Indent: First line: 0 ch, Line spacing: Double

Formatted: Font: (Default) Times New Roman, 12 pt

_The production function method is a metricmeasure obtained based on the definition of technical capacity. Technical capacity isrefers to the level of capacity at which all the capital stock, labor, land, and other factors of production invested in industrial production can be fully utilized for maximum output, assumingprovided that preferences and technologies are identified. In the actual application of the production function method for capacity measurement, theThe annual series of capacity data is estimated in the actual application of the production function method for capacity measurement by setting the production function and extrapolating the annual series of actual output to its “"boundary”" using the measurement method. TheIn this studypaper, the production function method is wais used in this study to calculate measure the capacity utilization rate of each industry and its three main product segments. The following are the reasons for using adopting this method are as follows. First,: (i) Tthethe production function method is based on neoclassical growth theory, which is not based ona product of historical experience and has a strong theoretical foundation basis, and the method has good validity, and reliability in measuring capacity utilization.; Second, (ii)unlikeCompared with the peak method, the production function method incorporates integrates the influence of technological progress, capital, and labor on output, as well as the degree of contribution of each production factor to output, which can reflect the changes in and characteristics of economic structure and production activities. Third,(iii)The the practical application of the production function method requires only requires data related to the three indicators of capital, labor, and output for practical application, and the production

function method has certain advantages over the cost function method in terms of data~~the~~ availability~~ of data compared with the cost function method~~. Finally,~~(iv)~~ given that~~As~~ different production links in each industry's ~~the~~ industrial chain~~ of each industry~~ involve different sub~~-~~sectors, ~~there are differences in~~ the maximum production capacity that each production link can achieve varies. Therefore, the production function approach can eliminate production capacity~~the~~ differences between~~in production capacity among~~ different enterprises in each production chain, making ~~the~~ measurements comparable and ~~also making the~~ estimation results directly applicable to the existing capacity assessment standards.

~~In this studypaper, the~~The Cobb-Douglas (C-D) production function is used in this study to model the production function in its basic form as follows:

$$\square_{\square,\square} = \square(\square_{\square,\square}, \square_{\square,\square}) = \square_{\square}\square^{\square}_{\square,\square}\square^{\square}_{\square,\square}\square^{-\square}, \square = 1,2,3, \square = 1,2,\ldots,\square$$

where i denotes~~is the~~ different links in the chain~~; and~~ t is the number of sample years; Y is the value of real output, expressed as annual ~~main~~ business revenue; $K_{i,t}$ is the fixed capital stock, expressed as annual average net fixed assets; $L_{i,t}$ is the labor input, which, using the standard followed by several scholars, is represented by~~, drawing on the practice of most scholars, using~~ the annual number of employees~~ as the labor input indicator~~; and A denotes the technology level, typically~~generally~~ used as a fixed constant. The parameters α and~~,~~ β denote the output elasticities of fixed capital stock and labor input, respectively.~~, and~~ A~~a~~ssuming constant returns to scale, we have:

Formatted: Indent: First line: 2.7 ch, Line spacing: Double
Formatted: Font: 12 pt
Formatted: Font: 12 pt
Formatted: Font: 12 pt
Formatted: Font: 12 pt
Formatted: Font: 12 pt
Formatted: Font: 12 pt
Formatted: Font: 12 pt
Formatted: Font: 12 pt
Formatted: Font: 12 pt
Formatted: Font: 12 pt
Formatted: Font: 12 pt
Formatted: Font: 12 pt
Formatted: Font: 12 pt
Formatted: Font: 12 pt
Formatted: Font: 12 pt
Formatted: Font: 12 pt
Formatted: Font: 12 pt
Formatted: Font: 12 pt
Formatted: Font: 12 pt
Formatted: Font: 12 pt
Formatted: Font: (Default) Times New Roman, 12 pt
Formatted: Indent: First line: 2.7 ch, Line spacing: Double
Formatted: Font: (Default) Times New Roman, 12 pt, Italic
Formatted: Font: (Default) Times New Roman, 12 pt
Formatted: Font: (Default) Times New Roman, 12 pt, Italic
Formatted: Font: (Default) Times New Roman, 12 pt
Formatted: Font: (Default) Times New Roman, 12 pt
Formatted: Font: (Default) Times New Roman, 12 pt, Italic
Formatted ...
Formatted: Font: (Default) Times New Roman, 12 pt
Formatted ...
Formatted ...
Formatted: Font: (Default) Times New Roman, 12 pt
Formatted ...
Formatted: Font: (Default) Times New Roman, 12 pt

$$0 < [illegible], [illegible] < 1 \text{ 且 } [illegible] + [illegible] = 1$$

The logarithm of the~~Logarithmizing the~~ above equation would be~~,~~:~~ ~~

$$[illegible]_{[illegible],[illegible]} = [illegible]_{[illegible],[illegible]} + [illegible]_{[illegible],[illegible]} + [illegible] - [illegible]$$

Extrapolating the production function to its ~~“~~"boundary,~~”~~", ~~we obtain~~ the boundary production function is obtained as:

$$[illegible]^{*}_{[illegible],[illegible]} = [illegible]_{[illegible],[illegible]} + [illegible]_{[illegible],[illegible]} + [illegible]$$

where $Y_{i,t}^{*}$ denotes the theoretical maximum level of output or~~and,~~ potential output.

Let $lnA = \alpha$ and $E(\mu) = \varepsilon$~~;~~, substituting these variables in~~to~~ the above equation:

$$[illegible]_{[illegible],[illegible]} = [illegible]_{[illegible],[illegible]} + [illegible]_{[illegible],[illegible]} + ([illegible] - [illegible]) - ([illegible] - [illegible])$$

Since $E(\mu - \varepsilon) = 0$, estimating~~estimation of~~ the above equation using ordinary least squares (OLS) yields:~~,~~

$$[illegible]_{[illegible],[illegible]} = \hat{[illegible]}_{[illegible],[illegible]} + \hat{[illegible]}_{[illegible],[illegible]} + ([illegible] - \hat{[illegible]})$$

The boundary production function generated by changing the constant term in the equation above is called~~The above equation is~~ the average production function, ~~which is the boundary production function obtained by adjusting its constant term,~~ and it is further treated as follows:

$$[illegible]([illegible]_{[illegible],[illegible]} - [illegible]_{[illegible],[illegible]}) = [illegible]\{[illegible]_{[illegible],[illegible]} - [\hat{[illegible]}_{[illegible],[illegible]} + \hat{[illegible]}_{[illegible],[illegible]} + ([illegible] - \hat{[illegible]})]\}$$

Thus, the boundary production function deduced from the estimation is:

$$\hat{[illegible]}_{[illegible],[illegible]} = [illegible]^{[illegible]} [illegible]^{[illegible]}_{[illegible],[illegible]} [illegible]^{[illegible]}_{[illegible],[illegible]}$$

Capacity utilization is measured as:

Formatted: Font: 12 pt
Formatted: Font: (Default) Times New Roman, 12 pt
Formatted: Font: (Default) Times New Roman, 12 pt, Font color: Black
Formatted: Line spacing: Double
Formatted: Font: 12 pt
Formatted ...
Formatted: Font: (Default) Times New Roman, 12 pt
Formatted: Line spacing: Double
Formatted: Font: 12 pt
Formatted ...
Formatted: Font: (Default) Times New Roman, 12 pt
Formatted: Line spacing: Double
Formatted ...
Formatted: Font: 12 pt
Formatted ...
Formatted ...
Formatted ...
Formatted: Font: 12 pt
Formatted ...
Formatted: Font: (Default) Times New Roman, 12 pt
Formatted: Line spacing: Double
Formatted: Font: 12 pt
Formatted ...
Formatted: Font: (Default) Times New Roman, 12 pt
Formatted: Line spacing: Double
Formatted ...
Formatted: Font: (Default) Times New Roman, 12 pt

$$\square\square = \square_{\square,\square}/\widehat{\square}_{\square,\square}$$

The capacity utilization rate estimated in this way has~~will have~~ a value of 1. In other words, the capacity utilization rate obtained for each listed company is not an absolute value.~~;~~ ~~100% of the~~The maximum capacity utilization rate ~~is the maximum value~~ of the selected sample during the period~~for~~ 2010~~–~~–2018 is 100%, while the remaining~~rest of the~~ estimates are relative to ~~values concerning~~ the maximum of the selected period. Because~~Since~~ the maximum capacity that~~ can be achieved by~~ each production link in the industry chain can achieve varies~~ is different~~, this treatment eliminates company~~the~~ differences~~ among companies~~ and makes the estimation results directly applicable to the existing capacity evaluation criteria.

Explanatory variable~~:~~

~~(2)~~ *~~the~~ D~~d~~egree of digitalization~~digitization~~ (DT)*~~:~~. Given that a standard metric to measure the level of firms'~~The current measurement of the degree of~~ digital transformation~~ of enterprises~~ is yet to be developed, ~~and~~ this study~~paper~~ uses Python to mine ~~the information on~~ digital transformation data from~~ involved in~~ the annual reports of listed companies. According to a~~the~~ review of existing studies on~~related to~~ digital transformation, enterprise~~the~~ digital transformation ~~of enterprises~~ is measured by capturing the use~~ application~~ of information technology such as the Internet by enterprises through text analysis [27]~~(Yang, Deming, and Liu, Yongwen, 2018)~~, but such indicators are unidimensional~~ the dimensions of such indicators are too single~~ and focus only on the application of a specific information technology, making~~which~~

Formatted: Font: 12 pt

Formatted: Font: 12 pt

Formatted: Font: 12 pt

Formatted: Font: 12 pt

Formatted: Font: 12 pt

Formatted: Font: 12 pt

Formatted: Font: (Default) Times New Roman, 12 pt

Formatted: Indent: First line: 2.7 ch, Line spacing: Double

Formatted: Line spacing: Double

Formatted: Font: (Default) Times New Roman, 14 pt, Bold

Formatted: Font: (Default) Times New Roman, 14 pt

Formatted: Font: (Default) Times New Roman, 12 pt, Italic

Formatted: Line spacing: Double, No bullets or numbering

Formatted: Font: (Default) Times New Roman, 12 pt

~~makes~~ it~~is~~ difficult to obtain a comprehensive ~~reflect the whole~~ picture of enterprise digitalization. This ~~Given this, this~~ study, however, creates~~paper builds~~ a relatively complete digital lexicon using~~with the help of~~ semantic representations of national digital economy policies ~~related to the digital economy,~~ and employs~~uses~~ machine learning-based text analysis methods to construct a more comprehensive indicator that reflects the degree of digitalization of Chinese listed enterprises. This digital lexicon is then expanded to a Chinese lexicon to conduct a~~Then,~~ text analysis ~~is conducted on the annual reports of listed companies by expanding the above digital lexicon to a Chinese lexicon, and then a text analysis is conducted~~ on the "~~"~~Management Discussion and Analysis"~~"~~ (MD&A) section of the listed companies' annual reports ~~of listed companies~~ based on machine learning to obtain the frequency of digital-related terms in the annual reports. Given that the~~Considering the difference in text~~ length of the MD&A sections of annual reports vary between companies, after extracting the frequency of each keyword in each listed company's annual report, ~~this study~~~~paper uses~~ the sum of the frequency of digitalization-related words divided by the length of the MD&A sections of annual reports is used to measure the degree of digitalization~~digitization~~ (DT) of micro-enterprises. This indicator is~~wais~~ multiplied by 100 for notational convenience. The higher the value of the DT indicator, the greater~~higher~~ the degree of digitalization~~digitization~~.

~~(3)~~ Mediating variables~~:~~

Formatted: Line spacing: Double

Formatted: Font: (Default) Times New Roman, 14 pt, Bold

Formatted: Font: (Default) Times New Roman, 14 pt

~~a. ~~*Firm innovation* (*Inn~~Inn~~*)~~:~~. The~~In this studypaper, the~~ data for corporate innovation in this study are primarily~~is mainly~~ derived from the number of invention patent applications in the CSMAR database's patent sub-database of listed companies and subsidiaries~~ in the CSMAR database~~, which is logarithmic processing to ensure data consistency. Data on~~related to~~ corporate innovation and innovation investments are~~were~~ compared to~~with~~ W~~W~~ind database data to identify~~check for~~~~and fill in~~ gaps, and the data are double-~~werewas~~ checked by consulting the companies' annual reports ~~of the companies~~ in the case of a few inconsistencies in the calibration process. Corporate~~The measurement of corporate~~ innovation is measured based on the Chinese patent database~~, mainly~~ due to the following reasons~~ considerations~~. First, it ensures~~:~~ ~~(i) Better~~ better data integrity~~,~~ as the CSMAR database matches with the Wind database to form long~~er~~-term panel data. Second, it ensures greater~~; (ii) Higher~~ data reliability~~,~~ as patent data is based on~~ the consideration of~~ corporate innovation behavior, as opposed to~~compared with~~ "~~"~~R&D investment"~~"~~ and "~~"~~new product output value.~~"~~"~~.~~ ~~(iii)~~ Finally, it provides a more~~More~~ detailed classification as it assists in distinguishing~~. Based on the information on different patent applications and licenses, it can better distinguish~~ between different types of enterprise innovation and refining~~refine~~ enterprise innovation behavior based on information from different patent applications and licenses. It is noteworthy~~ needs to be further explained~~ that patent data contains additional information, such as~~ contains more elements, including~~ application information, authorization~~ information~~, and patent citation information. Given~~Considering~~ that this study~~paper~~ focuses on the impact of digital transformation

Formatted: Font: (Default) Times New Roman, 12 pt, Italic

Formatted: Font: (Default) Times New Roman, 12 pt, Italic

Formatted: Indent: First line: 0 ch, Line spacing: Double

Formatted: Font: (Default) Times New Roman, 12 pt

Formatted: Font: (Default) Times New Roman, 12 pt

on innovation behavior, ~~its focus should be reflected in~~ the fluctuation in~~of~~ the number of patent applications should reflect this focus; thus,~~ therefore,~~ ~~based on which we adopt~~ the number of ~~invention~~ patent applications for inventions are used to measure innovation behavior.

~~b~~ b. *Degree of production specialization (VSI)*:~~.~~ Vertical~~In studies on the division of labor, vertical~~ integration and specialization are diametrically opposed~~ opposite~~ concepts in labor studies, with higher vertical integration implying lower specialization~~,~~ and vice versa. Value added to sales (VAS), proposed by Adelman [28] as~~is~~ a proxy measure of vertical integration~~, in academia, which was first proposed by Adelman (1955) and~~ has been widely used in management~~subsequent~~ studies thus far. The basic idea is to estimate the ~~ratio of~~ value-added to sales revenue ratio;~~,~~ ~~and~~ the higher the ratio, the greater the firm's~~higher the level of~~ vertical integration~~ of the firm~~. Following previous studies [29,30],~~Referring to the studies of Buzzell (1983), Fan Ziying, and Peng Fei (2017),~~ this study~~paper~~ uses a~~adopts the~~ modified value-added method to estimate VAS ~~for the metric. The specific calculations~~ were~~is~~ as follows:~~,~~

$$\mathrm{VAS} = \frac{\text{Value added} - \text{net profit after tax} + \text{normal profit}}{\text{Income from main business} - \text{net profit after tax} + \text{normal profit}}$$

$$= \frac{\text{Value Added } - \text{Net Income After Tax} + \text{Net Assets} \times \text{Average Net Asset Margin}}{\text{Income from main business} - \text{net income after tax} + \text{net assets } \times \text{Average net asset margin}}$$

Formatted: Line spacing: Double

Formatted: Font: (Default) Times New Roman, 12 pt, Italic

Formatted: Font: (Default) Times New Roman, 12 pt

Formatted: Font: (Default) Times New Roman, 12 pt, Italic

Formatted: Font: (Default) Times New Roman, 12 pt

Formatted: Indent: First line: 0 ch, Line spacing: Double

Formatted: Line spacing: Double

Value added is expressed as the difference between the firm's sales and purchases, and VAS measures value_-added as a percentage of sales revenue, with larger values ~~of this metric~~ indicating greater~~higher levels of~~ vertical integration. Following Zhang [31]~~Drawing on Zhang's (2004) study~~, the inverse of VAS is defined as the degree of firm specialization (VSI). Therefore, VSI is calculated as:~~The details are as follows,~~:

$$\text{[illegible]} = 1 - \text{[illegible]}$$

A~~Among them, a~~ higher~~larger value of the~~ VSI value ~~metric~~ indicates a higher level of enterprise specialization. Following Fan Ziying and Peng Fei [30], ~~To ensure the validity of the metric, referring to the study of Fan Ziying and Peng Fei (2017), the~~ observations in which the VSI deviates from the reasonable value domain (~~[~~0,1)~~]~~ interval are excluded from the analysis to ensure the metric's validity~~in this studypaper~~.

~~(4)~~ Other variables~~.~~

Many~~In reality, many~~ factors contribute~~lead~~ to firm overcapacity~~ in the firm~~. In addition to the digital transformation of firms, ~~which is the focus of this study,~~ characteristics such as firm size (*size*), gearing (*lev*), return on total assets (*roa*), and~~as well as~~ geographical and industry characteristics, such as the nature of firms (*soe*), can have an impact on~~affect~~ a~~the~~ firm's capacity utilization. Therefore, this study controls for the~~se above~~ variables~~ weare controlled for in this studypaper~~. Furthermore, *code*~~In addition, code~~, *industry*, *province*, and *year* denote firm, industry, region, and

Formatted: Font: (Default) Times New Roman, 12 pt
Formatted: Indent: First line: 2.7 ch, Line spacing: Double
Formatted: Font: 12 pt
Formatted: Centered
Formatted: Font: (Default) Times New Roman, 12 pt
Formatted: Line spacing: Double
Formatted: Font: (Default) Times New Roman, 14 pt, Bold
Formatted: Line spacing: Double
Formatted: Font: (Default) Times New Roman, 14 pt
Formatted: Font: (Default) Times New Roman, 12 pt
Formatted: Indent: First line: 0.39", Line spacing: Double
Formatted: Font: (Default) Times New Roman, 12 pt, Italic
Formatted: Font: (Default) Times New Roman, 12 pt
Formatted: Font: (Default) Times New Roman, 12 pt, Italic
Formatted: Font: (Default) Times New Roman, 12 pt
Formatted: Font: (Default) Times New Roman, 12 pt, Italic
Formatted: Font: (Default) Times New Roman, 12 pt
Formatted: Font: (Default) Times New Roman, 12 pt, Italic
Formatted: Font: (Default) Times New Roman, 12 pt
Formatted: Font: (Default) Times New Roman, 12 pt, Italic
Formatted: Font: (Default) Times New Roman, 12 pt
Formatted: Font: (Default) Times New Roman, 12 pt, Italic
Formatted: Font: (Default) Times New Roman, 12 pt
Formatted: Font: (Default) Times New Roman, 12 pt, Italic
Formatted: Font: (Default) Times New Roman, 12 pt
Formatted: Font: (Default) Times New Roman, 12 pt, Italic
Formatted: Font: (Default) Times New Roman, 12 pt

year fixed effects, respectively. Table 1 summarizes the definitions of all the key variables used in this study.

Table 1. Variable definitions

~~Variable definition~~

Variable type~~Types~~	Name	Symbols	Measurements
Explained variable	Capacity utilization rate	*CU*	Actual~~Find the actual~~ ~~output~~ and pote[illegible] calculated using ~~by~~ the prod[illegible] ~~using~~(revenue from main business / ma[illegible] revenue)
Explanatory variable	Digitalization of enterprises	*DT*	Text analysis of listed companies' a[illegible] ~~listed companies~~ using Python to estim[illegible] ~~of~~ the degree of digital transformation
Mediating ~~Intermediate~~ variables	Corporate innovation	*Inn*	Logarithm of the number of i[illegible] applications by listed companies in the y[illegible]
	Production specialization~~Professional production~~	*VSI*	The modified value-added method is u[illegible] firm's value-added ~~of the firm~~ as a prop[illegible] ~~of~~ sales revenue, measuring ~~and this me~~[illegible] level~~degree~~ of vertical integration ~~of th~~[illegible] inverse (1-VAS) of which ~~whose~~ i[illegible] ~~1-VAS~~ indicates the level ~~degree~~ of spec[illegible]
Control variables	Company scale	*size*	ln(Number of employees)
	Gearing ratio	*lev*	(Total liabilities at the end of period / [illegible] 100%
	Total return on assets	*roa*	(Total profit – (income tax rate × [illegible] (average total liabilities + average own[illegible] 100%
	Nature of business property	*soe*	If the company is a state-owned enterpri[illegible] 1; ~~;~~ otherwise, it is 0
	Age of the company	*age*	ln(Year of observation+1 - Year of regis[illegible]
Fixed effects	Region	*province*	Province of the company
	Industry	*industry*	Industry categories
	Year	*year*	Current year
	Company	*code*	Stock code of listed companies

~~3.~~ Empirical model~~-construction~~

Following previous studies [21,31],

~~Evaluating~~ the impact of digital transformation (inverted "U" mechanism), production specialization, enterprise innovation, and capacity utilization are evaluated using~~,~~

based on the studies of Bing-Zhan Shi and Jian-Tong Li (2020) and Chun Yuan et al. (2021), the inverted "U" mechanism of digital transformation, as well as the path of action on capacity utilization and boundary conditions on enterprise capacity utilization were analyzed based on theoretical analysis. To test the study's hypotheses, the The impact of digital transformation on capacity utilization To this end, the quadratic terms of the explanatory variables are incorporated, and the following models (1) to (2) are developed:

to test hypotheses H1 and H2.

□□$_{□,□}$ = □$_0$ + □$_1$□□$_{□,□}$ + □□□□□□□□□ + □$_{□,□}$

(1)

□□$_{□,□}$ = □$_0$ + □$_1$□□$_{□,□}$ + □$_2$□□$^2_{□,□}$ + □□□□□□□□□ + □$_{□,□}$

(2)

If the coefficients α_1 and α_2 are significantly positive, it meansindicates that the enterprise's digital transformation of the enterprise is positively promotesing itsthe enterprise's capacity utilization, and the enterprise's capacity is gradually and rapidly increasesing in tandem with the depth of digital transformation.; However, ifotherwise, the coefficients α_1 isare significantly positive and α_2 isare significantly negative, it meanswhich indicates that the impact of the enterprise's digital transformation of the enterprise on its capacity utilization is inverted U-shaped, thereby supportingand the theoretical hypothesis H2 holds. If the coefficient α_2 is insignificant, it meansindicates

that the impact of enterprises'the digital transformations of enterprises on their capacity utilization is linear.

FollowingReferring to Zhonglin Wen and Baojuan Ye [32]'s (2004) study on the mediation model, the effects of digital transformation, production specialization, firm innovation, and capacity utilization are tested by usingincorporating mediating variables. Hypothesis, and hypothesis H1 is tested using the followingthrough models (3) to (7). The specific models are as follows:

:

[illegible] = [illegible] + [illegible] + [illegible] + ∑ [illegible] + ∑ [illegible] + [illegible] （3）

[illegible] = [illegible] + [illegible] + β [illegible] + [illegible] + ∑ [illegible] + ∑ [illegible] + [illegible] （4）

[illegible] = [illegible] + [illegible] + [illegible] + ∑ [illegible] + ∑ [illegible] + [illegible] （5）

[illegible] = [illegible] + [illegible] + β [illegible] + [illegible] + ∑ [illegible] + ∑ [illegible] + [illegible] （6）

[illegible] = [illegible] + [illegible] + θ [illegible] + β [illegible] + [illegible] + ∑ [illegible] + ∑ [illegible] + [illegible] （7）

ResultsIV. Analysis of empirical results

1. Descriptive analysis

As shown inIn Table 2, the descriptive statistics of the main variables show that the mean and standard deviation of capacity utilization level (*CU*) are 1.855 and 5.157, respectively, with a maximum value of 219.8 and a minimum value of 0.02, indicating that the degree of capacity utilization varies significantly betweengreatly among enterprises. *DT* has aThe mean and standard deviation of DT are 0.229 and 0.353, respectively, with a median, maximum, and minimum values value of 0.082,

Formatted ...

Formatted: Line spacing: Double

Formatted: Font: 12 pt

Formatted ...

Formatted ...

Formatted ...

Formatted ...

Formatted ...

Formatted: Font: (Default) Times New Roman, 18 pt

Formatted: Line spacing: Double

Formatted: Font: (Default) Times New Roman, 16 pt, Bold

Formatted: Font: (Default) Times New Roman, 12 pt

Formatted: Indent: First line: 2.7 ch, Line spacing: Double

Formatted ...

~~and maximum and minimum values of~~ 3.637, and 0, respectively. This~~ individually, which~~ also shows~~indicates~~ that the degree of digitalization varies significantly between~~ widely among~~ enterprises. The mean and variance of enterprise specialization (*VSI*) are 0.549 and 0.212, respectively, with ~~and the~~ maximum and minimum values of~~ value is~~of 1 and~~ the minimum value is~~of 0, respectively, indicating the significant differences in the level~~ which indicates that there are large differences in the degree~~ of specialization of different enterprises. ~~In addition~~Furthermore, ~~the~~ statistical results by industry show that there are considerable~~large~~ differences between industries in terms of digitalization~~ in the~~ development with~~ of digitalization in each industry, and the overall presentation of~~ the following characteristics.~~:~~ (1)~~a.~~ rapid development of digitalization in the service sector; (2)~~b. The trend of~~ better integration of manufacturing and digital technology~~, which~~ is especially noticeable~~ particularly prominent~~ in technology-intensive industries; (3)~~.;~~ ~~c. Digital transformation in~~ agriculture, public utilities, and resource-intensive traditional industries have significantly lower~~ is much less than the average industrial~~ digital transformation than the general industry average. Furthermore~~Moreover~~, there are considerable differences in~~ the level of~~ capacity utilization across industries, with~~ shows large differences, where~~ more~~higher~~ capital-intensive industries having~~have~~ lower capacity utilization and~~,~~ technology-intensive industries having~~have~~ higher capacity utilization~~, and there are large differences in capacity utilization between industries~~. This is consistent with the conventional view of overcapacity.

Formatted: Font: (Default) Times New Roman, 12 pt, Italic

Formatted: Font: (Default) Times New Roman, 12 pt

Table 2.~~–~~

Descriptive statistics results

variable	N	mean	p50	Sd	min	max	range
CU	15942	1.855	0.960	5.157	0.0190	219.8	219.8
DT	15942	0.229	0.0820	0.353	0	3.637	3.637
DT^2	15942	0.177	0.00700	0.570	0	13.23	13.23
Inn	10766	2.260	2.197	1.463	0	9.108	9.108
VSI	15942	0.549	0.561	0.212	0	1	1
roa	15942	0.0400	0.0380	0.0750	-1.648	4.837	6.485
size	15942	7.710	7.616	1.324	2.565	13.22	10.66
lev	15942	0.427	0.420	0.211	0.00700	0.995	0.988
age	15942	2.057	2.197	0.894	0	3.367	3.367
soe	15942	0.398	0	0.489	0	1	1

~~2.~~ Baseline regression results

Table 3 presents the ~~reports the results of the~~ benchmark regression results~~s~~, where the first column controls only for fixed effects and the second column includes~~adds~~ firm- and region-level control variables. The results show that the *DT* coefficients ~~on DT~~ are all significantly positive at ~~the level of~~ less than 1%, implying that the greater~~higher~~ the degree of digitalization, the higher the~~its~~ capacity utilization. In terms of economic significance, the results in the second column~~, for example,~~ show that a 1%~~one percent~~ increase in a firm's~~the~~ degree of digital transformation increases~~ of a firm will increase~~ its capacity utilization by 31.5%. This suggests that, both economically and statistically, digital transformation has~~ does have~~ a significant incentive effect on enterprise capacity utilization,~~ both in an economic and statistical sense,~~ and hypothesis H1 is supported by these empirical results. As stated ~~mentioned~~ in the previous theoretical analysis, firm capacity utilization is determined by the difference~~ of firms depends on the gap~~ between actual output and ~~its~~ potential

Formatted: Indent: First line: 2.7 ch

Formatted: Font: (Default) Times New Roman, 12 pt, Not Highlight

Formatted: Font: (Default) Times New Roman, 12 pt, Bold, Not Highlight

Formatted: Indent: First line: 0 ch

Formatted: Font: 12 pt, Bold, Not Highlight

Formatted: Font: (Default) Times New Roman, 16 pt, Bold

Formatted: Line spacing: Double

Formatted: Font: (Default) Times New Roman, 12 pt

Formatted: Indent: First line: 2.7 ch, Line spacing: Double

Formatted: Font: (Default) Times New Roman, 12 pt, Italic

Formatted: Font: (Default) Times New Roman, 12 pt

maximum output, and digital transformation of firms may either increase the potential output, ~~thus~~ exacerbating the overcapacity situation, or increase the actual output level, thereby~~thus~~ enhancing ~~the~~firms' capacity utilization~~ of firms~~. The results in Table 3 show that~~,~~ digital transformation has a significantly greater~~higher~~ effect on ~~the~~ actual output than ~~on its~~ potential output, and that enterprise~~the~~ digital transformation ~~of enterprises~~ significantly increases ~~the~~ capacity utilization level, supporting hypothesis H1 in the theoretical model. To verify the validity of ~~whether~~ hypothesis H2~~ is valid or not~~, ~~this paper performs~~ a quadratic treatment on the explanatory variables is performed to obtain the variable *DT2*~~,~~. The findings~~and the results~~ in Table 3's~~the~~ third column~~–~~ show that the ~~*coefficient of*~~ *DT2* ~~is~~coefficient is significantly negative, and when combined with the coefficient of the primary term, it becomes evident~~is clear~~ that the impact~~effect~~ of digital transformation on capacity utilization is nonlinear and inverted U-shaped.

Table 3.

Benchmark regression results

Variables	(1) CU	(2) CU	(3) CU
DT	0.315**	0.437**	0.93***
	(0.137)	(0.18)	(0.338)
DT^2			-0.283*
			(0.164)
size2		-1.053***	-1.054***
		(0.065)	(0.065)
roa		1.936***	1.923***
		(0.432)	(0.432)
lev		2.693***	2.703***
		(0.311)	(0.311)
age		-0.34***	-0.345***
		(0.106)	(0.106)
soe		-0.141	-0.138

Formatted: Font: (Default) Times New Roman, 12 pt, Italic

Formatted: Font: (Default) Times New Roman, 12 pt

Formatted: Font: (Default) Times New Roman, 12 pt, Italic

Formatted: Font: (Default) Times New Roman, 12 pt

Formatted: Indent: First line: 2.7 ch

Formatted: Font: (Default) Times New Roman, 12 pt, Bold

Formatted: Font: (Default) Times New Roman, 12 pt, Bold, Not Highlight

Formatted: Font: (Default) Times New Roman, 12 pt, Not Highlight

Formatted: Font: (Default) Times New Roman, 12 pt, Bold, Not Highlight

Formatted: Font: 12 pt, Bold, Not Highlight

Formatted: Indent: First line: 0 ch

	(1)	(2)	(3)
		(0.24)	(0.24)
Constants	1.727***	9.406***	9.359***
	(0.087)	(0.516)	(0.517)
Observations	15942	15912	15912
Adj-R^2	0.673	0.673	0.673

Note: t-statistics adjusted for firm-level clustering are in parentheses;~~,~~ ***, **, and * indicate significance~~t~~ at the 1%, 5%, and 10% levels, respectively, and the same as in the following tables, if not otherwise specified.

~~3.~~ Robustness tests

~~(1)~~ Endogenous problems

Previous studies may have raised endogeneity concerns for~~issues, which could be due to~~ the following two reasons: (a)~~ a.~~ i~~I~~ncreased digital transformation of enterprises will improve their production and business environment, as well as~~ and~~ promote higher levels of capacity utilization; (b)~~ b.~~ i~~I~~ncreased capacity utilization may also necessitate~~have a more pressing need for the~~ high-level digital development of enterprises, weakening unnecessary trade resistance~~,~~ and assisting~~ helping~~ them in improving~~ better improve their~~ production efficiency. The~~ ~~ instrumental variables approach is used in this study to~~To~~ mitigate ~~as much as possible~~ the reverse causality of the aforementioned~~this~~ potential endogenous effect on the findings~~ of this study,~~. ~~the instrumental variables approach is used to test this paper.~~

Following~~Referring to the study by Qunhui~~ Huang et al. [34]~~ (2019)~~, the instrumental variables for firm digitalization~~ the digitization of firms~~ in this study are based on~~paper use~~ 1984 postal data for each city. This is done primarily because~~The main reason is that~~ the communication methods used in the previous~~past~~ development process in the firm's location ~~of the enterprise~~ will influence~~affect~~ the firm's

Formatted: Font: (Default) Times New Roman, 16 pt, Bold

Formatted: Font: (Default) Times New Roman

Formatted: Font: (Default) Times New Roman, 16 pt, Bold

Formatted: Line spacing: Double

Formatted: Font: (Default) Times New Roman, 14 pt, Bold

Formatted: Font: (Default) Times New Roman, 12 pt

Formatted: Indent: First line: 2.7 ch, Line spacing: Double

Formatted: Indent: First line: 2.7 ch, Line spacing: Double

Formatted: Font: (Default) Times New Roman, 12 pt

application and acceptance of information technology in terms of technology level and social preferences during by the enterprise in the sample period in terms of technology level and social preferences, satisfyingwhich satisfies the correlation condition; meanwhile. Postal, postal and telecommunications, as social infrastructure, primarily mainly provide communication services to the population and do not directly influence firms' act on the capacity utilization processes, thereby satisfyingprocess of enterprises and, satisfy the exogeneity condition. Furthermore, becauseIn addition, considering that the 1984 post and telecommunications information for of each city is cross-sectional data that, which is difficult to usbe directly used as an instrumental variable for panel data, this study follows referring to the method of Yuan Chun et al. [21] and(2021), this studypaper uses the cross-product term of the number of national Iinternets accesses with a lag of one period and the number of fixed telephones per 10,000 people at the end of 1984 Ger as an instrumental variable for the digitalization progress of enterprises in the current period., and reports the instrumental variable method in the following table second stage regression results. The results in column (1) of Table 4 show that the coefficients of the explanatory variable coefficientss are significantly positive at the 5% level, thereby demonstrating the validity of the study's primary findings; therefore,, so the main conclusions of this studypaper still hold.

(2) Robustness test

a. Alternative measurestests of digital transformation.

Formatted: Font: (Default) Times New Roman, 12 pt

Formatted: Indent: First line: 2.7 ch, Line spacing: Double

Formatted: Font: (Default) Times New Roman, 14 pt

Formatted: Line spacing: Double

Formatted: Font: (Default) Times New Roman, 14 pt, Bold

Formatted: Font: (Default) Times New Roman, 14 pt

Formatted: Font: (Default) Times New Roman, 14 pt, Bold

Formatted: Font: (Default) Times New Roman, 14 pt

,Several methods are employed inIn this study to arrive at, various methods werepaper, a variety of methods is used to perform alternative measures of a company's digital transformation. TheSince the quantification of digital transformation in the sample firms is based on of the above-mentioned enterprises only involves the frequency of special words ofwith a digital nature appearing in the firms' annual statements of the enterprise, which reflects the importance of the firms' digital transformation of the enterprise. The quantification, however,, and does not consider firms'involve the investment in the developmentconstruction of the digital infrastructure of the enterprise. Therefore, by using digital technology applications and digital technology devices as proxy variables for enterprise digital transformation in the robustness test,in the process of robustness testing, this studypaper strengthens the examination of the conditions under which digital transformation can be achieved by using digital technology applications and digital technology devices as proxy variables for the enterprises' digital transformation. Specifically, they represent the number of people employedengaged in information transmission, computer services, and the software industry /(10 million,000,000), and the number of Internet broadband access subscribers /(100 million) in the year in the city where the listed company is located, respectively. According to the results shown in columns (2) and (3) of Table 4, the coefficients of the explanatory variable coefficientss are significantly positive, indicating thatso the results of this studypaper remain robust. Particularly,, especially the degree of digital transformation, which has been replaced by the number of Internet broadband access subscribers, has a significantwhose coefficient is significant

Formatted: Font: (Default) Times New Roman, 12 pt

at the 1% level, indicating that the direction and significance of the regression coefficients remain the same as previously stated, thereby evidencing the robustness of the primary findings ~~above, that isi.e., they pass the robustness test~~.

Robustness tests~~.~~

Excess~~In this studypaper, the opposite variable of capacity utilization, excess~~ capacity (*OC*), the inverse variable of capacity utilization, is~~wais~~ used ~~–~~to replace the explanatory variable as an~~for the~~ alternative measure. Column~~The results in column~~ (4) of Table 4 shows that the ~~coefficient of the~~ explanatory variable coefficient is significantly negative, and because~~since~~ the alternative measure is an inverse variable~~a negative mapping~~ of capacity utilization, this indicates that digitalization~~digitization~~ significantly suppresses corporate overcapacity and supports the robustness of the study's primary findings ~~basic conclusion of the studypaper as robust~~. Regional~~In column (5), this study adds regional~~ and industry fixed effects are included in column (5), which are~~that isi.e., adding~~ the interaction fixed effects of province and year, and industry ~~and industry,~~ and year of listed companies. The ~~results show that the coefficients of the~~ explanatory variable coefficients~~s~~ are significant at the 5% level, thereby proving the robustness of the primary~~; thus,, so the main~~ findings ~~of this studypaper are robust~~.

Table 4.~~–~~

Results of robustness tests

Variables	(1) CU	(2) CU	(3) CU	(4) OC	(5) CU
DT_w1	4.567**			-0.099*	0.469**

Formatted: Indent: First line: 0 ch, Line spacing: Double

Formatted: Font: (Default) Times New Roman, 14 pt, Bold

Formatted: Line spacing: Double

Formatted: Font: 14 pt, Bold, Not Highlight

Formatted: Font: (Default) Times New Roman, 12 pt

Formatted: Font: (Default) Times New Roman, 12 pt, Italic

Formatted: Font: (Default) Times New Roman, 12 pt

Formatted: Indent: First line: 0 ch

Formatted: Font: (Default) Times New Roman, 12 pt, Not Highlight

Formatted: Font: (Default) Times New Roman, 12 pt, Bold, Not Highlight

Formatted: Font: 12 pt, Bold, Not Highlight

Formatted: Font: (Default) Times New Roman, 12 pt, Bold, Not Highlight

	(2.106)			(0.055)	(0.198)
news		0.009*			
		(0.005)			
Int us			10.526***		
			(3.068)		
size2	-0.596***	-0.997***	-0.992***	0.865***	-1.043***
	(0.038)	(0.071)	(0.071)	(0.024)	(0.071)
roa	3.818***	1.668***	1.66***	-1.183***	1.785***
	(0.603)	(0.444)	(0.444)	(0.109)	(0.444)
lev	4.818***	2.612***	2.641***	-0.15	2.707***
	(0.275)	(0.33)	(0.329)	(0.095)	(0.329)
age	0.258***	-0.341***	-0.342***	0.113***	-0.334***
	(0.067)	(0.118)	(0.118)	(0.032)	(0.117)
soe	0.195	-0.114	-0.106	-0.093	-0.277
	(0.135)	(0.265)	(0.265)	(0.075)	(0.255)
Constants	2.614***	9.015***	8.743***	13.455***	9.363***
	(0.47)	(0.568)	(0.575)	(0.176)	(0.565)
Observations	15087	14751	14751	8161	15909
Adj-R^2	0.061	0.69	0.691	0.802	0.686

~~V.~~ Additional Analyses~~Further Analysis~~

~~1.~~ Mechanism testing and analysis

The~~Previous~~ analyses thus far reveal ~~show~~ that the mechanism of digital transformation to promote enterprise capacity utilization is to promote enterprise innovation and production specialization, which, in turn, increases the efficiency of enterprises' ~~raw~~ resource utilization. Therefore, ~~this studypaper introduces~~ the degree of production specialization (*VSI*) and technological innovation are included in the~~into the~~ analysis and ~~and proposes~~ hypothesis H1 is proposed, the~~whose~~ regression results of which are shown in Table 5.

Table 5–

Heterogeneity analysis results

Formatted: Font: (Default) Times New Roman, 18 pt
Formatted: Line spacing: Double
Formatted: Font: (Default) Times New Roman, 16 pt
Formatted: Font: (Default) Times New Roman, 16 pt, Bold
Formatted: Font: (Default) Times New Roman, 12 pt
Formatted: Indent: First line: 2.7 ch, Line spacing: Double
Formatted: Font: (Default) Times New Roman, 12 pt, Italic
Formatted: Font: (Default) Times New Roman, 12 pt
Formatted: Indent: First line: 0 ch
Formatted: Font: (Default) Times New Roman, 12 pt, Not Highlight
Formatted: Font: (Default) Times New Roman, 12 pt, Bold, Not Highlight
Formatted: Font: 12 pt, Bold, Not Highlight
Formatted: Font: (Default) Times New Roman, 12 pt, Bold, Not Highlight

	(1)	(2)	(3)	(4)	(5)
	VSI	CU	Inn	CU	CU
DT	0.023***	0.399**	0.209***	0.102	0.088
	(0.007)	(0.18)	(0.05)	(0.064)	(0.064)
VSI		1.633***			0.71***
		(0.221)			(0.087)
Inn				0.07***	0.069***
				(0.014)	(0.014)
size	0.002	-1.056***	0.321***	-0.857***	-0.851***
	(0.003)	(0.065)	(0.021)	(0.028)	(0.028)
roa	0.145***	1.698***	0.434**	4.53***	4.316***
	(0.017)	(0.432)	(0.212)	(0.273)	(0.273)
lev	0.122***	2.494***	0.223**	1.735***	1.612***
	(0.012)	(0.311)	(0.096)	(0.123)	(0.124)
age	0.002	-0.343***	0.021	-0.008	-0.015
	(0.004)	(0.106)	(0.031)	(0.04)	(0.04)
soe	-0.018**	-0.111	-0.077	-0.121	-0.1
	(0.009)	(0.24)	(0.067)	(0.086)	(0.086)
Constants	0.473***	8.633***	-.406**	7.115***	6.752***
	(0.02)	(0.526)	(0.166)	(0.214)	(0.217)
Observations	15912	15912	10589	10589	10589
Adj-R^2	0.708	0.674	0.806	0.855	0.856

The results in column (1) of Table 5 show~~indicate~~ that enterprise digital transformations~~ of enterprises~~ are significantly and positively related to production specialization at the 1% level, which can be interpreted~~ may be seen~~ as moderate digital transformation assisting~~helpings~~ enterprises to exploit their comparative advantages in resources and technology, improve~~s~~ their transaction environment, reduce~~s~~ their internal control costs, and promote~~s~~ their specialization. Column (2) demonstrates~~ shows~~ that the ~~*coefficient of*~~ *VSI* coefficient is significantly positive at the 1% level, whereas~~while~~ the *DT* coefficient ~~of DT~~ decreases in both ~~in~~ value and significance. Thus, the combined effect test using the stepwise regression method confirms~~ verifies~~ that digital transformation improves capacity utilization by

Formatted: Font: (Default) Times New Roman, 12 pt

Formatted: Indent: First line: 2.7 ch, Line spacing: Double

Formatted: Font: (Default) Times New Roman, 12 pt, Italic

Formatted: Font: (Default) Times New Roman, 12 pt

Formatted: Font: (Default) Times New Roman, 12 pt, Italic

Formatted: Font: (Default) Times New Roman, 12 pt

encouraging~~promoting~~ firms to achieve production specialization ~~specialize in production~~. Column (3) shows~~manifests~~ that at the 1% level, firm digital transformation ~~of firms~~ significantly promotes technological innovation ~~of firms at the 1% level~~, probably because digital transformation stimulates product and process innovation, which in turn promotes firms’ technological innovation ~~of firms~~. Column (4) shows that the coefficient of firms’ technological innovation ~~of firms~~ is significantly positive at the 1% level, but the coefficient of digital transformation decreases in value and is not significant at all, indicating~~which indicates~~ that the effect of *DT* on firms’~~the~~ capacity utilization ~~of firms~~ is ~~basically~~ translated into technological innovation ~~of firms, and~~ and thus there is technological innovation ~~to~~ promoting~~e~~ the capacity utilization of firms. Such technological innovation~~s~~ has~~have had~~ a well-mediated impact ~~effect~~. Column (5) incorporates ~~puts~~ all mediating variables into the model, and the comparison reveals that the ~~*coefficient of*~~ *VSI* coefficient is significantly larger than the coefficient~~that~~ of technological innovation for the two mediating variables that are also significant at the 1% level.~~,~~ T~~w~~his~~ch~~ indicates that both increased ~~the improvement of firms'~~ specialized production capacity and technological innovation can positively influence ~~promote~~ firms' capacity utilization, but the effect of specialized production capacity is stronger~~larger~~. However,~~ByIn contrast,~~ when~~comparing the coefficients of~~ the explanatory variable coefficients (*DT*) are compared, the effect of digital transformation is not significant, despite having~~although it has~~ a positive impact on~~relationship with promoting~~ capacity utilization.

Formatted: Font: (Default) Times New Roman, 12 pt, Italic

Formatted: Font: (Default) Times New Roman, 12 pt

Formatted: Font: (Default) Times New Roman, 12 pt, Italic

Formatted: Font: (Default) Times New Roman, 12 pt

Formatted: Font: (Default) Times New Roman, 12 pt, Italic

Formatted: Font: (Default) Times New Roman, 12 pt

2. Heterogeneity tests grouping test

As discussed in the previous theoretical analysis, digitalization alterswill change the internal ecology and external environments of enterprises, affectingwhich will affect their capacity utilization level. Specifically, digitalizationdigitization improves enterprises' capacity utilization levelthe capacity utilization level of enterprises by promoting innovation and specialized production specialization. However, the levels of enterprise capacity utilization and the digital transformation of enterprises varyies across enterprises and industries, which may result in lead to heterogeneity in the effect of digital transformation on enterprisethe capacity utilization of enterprises. ThereforeTo this end, this study's sample ispaper further subdivideds the sample to examine the performance of heterogeneity based on corporate governance level, companycorporate size, and industry dimensions.

a. Heterogeneity analysis based on enterprise size

Columns (1) and (2) of Table 6 presentgive the estimation results for the two sub-samples: of firms with less than average capital size and firms with greater than average capital size, respectively. The It can be seen that the estimated coefficient of *DT* in the sample of firms with the smaller capital size is significant at the 1% level. However, the regression results forin the sample of firms with larger capital are not significant, and the coefficients are negative. This result suggests that the digital transformation of firms with smaller capital size firms can effectively contribute to anthe increaserise in their capacity utilization levels by stimulating firm innovation

Formatted: Font: (Default) Times New Roman, 16 pt, Bold

Formatted: Font: (Default) Times New Roman, 16 pt, Bold

Formatted: Line spacing: Double

Formatted: Font: (Default) Times New Roman, 16 pt

Formatted: Font: (Default) Times New Roman, 12 pt

Formatted: Indent: First line: 2.7 ch, Line spacing: Double

Formatted: Indent: First line: 0 ch

Formatted: Font: (Default) Times New Roman, 14 pt, Bold

Formatted: Line spacing: Double, No bullets or numbering

Formatted: Font: (Default) Times New Roman, 12 pt

Formatted: Indent: First line: 2.7 ch, Line spacing: Double

Formatted: Font: (Default) Times New Roman, 12 pt, Italic

Formatted: Font: (Default) Times New Roman, 12 pt

and enhancing specialized production specialization. However, in firms with larger capital, the digital transformation has no significant effect on of firms suppresses their capacity utilization levels in firms withof larger capital sizes, with no significant effect. There may be more resistance to alleviating their overcapacity in such enterprises with larger capital sizes, which reduces the impact ofis not conducive to digital transformation playing its powerful role. Therefore, larger capital-intensive firms find it challenging to improveit is difficult for larger capital-intensive enterprises to enhance their capacity utilization through digital transformation.

b. Heterogeneity analysis based on corporate governance level

Columns (3) and (4) of Table 6 presentgive the results of the sub-sample regression resultss foron firms with two different levels of governance. TheIt can be seen that the estimated *DT* coefficient of DT in the sample with poorer governance is significantly positive at the 1% level. Although numerically positive, the The regression results forin the sample with higher levels ofbetter governance levels are not significant, although they are still numerically positive. This suggestsIt indicates that firms with lower levels of governance will benefit from the digital transformation of firms in firms with poorer levels of governance will contribute to their capacity utilization by stimulating innovation and improvingenhancing specialized production, which in turn will lead to an increase in their capacity utilization. DigitalFor poorly governed firms, digital transformation can compensate for deficitswasted in capacity utilization caused by due to athe lack of corporate governance in poorly governed firms by improving their resource allocation capabilities. Conversely, the impactFor

Formatted: Indent: First line: 0 ch

Formatted: Font: (Default) Times New Roman, 14 pt, Bold

Formatted: Line spacing: Double, No bullets or numbering

Formatted: Font: (Default) Times New Roman, 12 pt

Formatted: Indent: First line: 2.7 ch, Line spacing: Double

Formatted: Font: (Default) Times New Roman, 12 pt, Italic

Formatted: Font: (Default) Times New Roman, 12 pt

~~better governance, the effect~~ of digital transformation on ~~promoting~~ capacity utilization is not significant in firms with a sound governance system.

This could~~The reason may~~ be because~~that~~ companies with higher governance levels have greater~~higher~~ resource allocation capabilities, making~~and~~ the effect of further improvement in capacity utilization levels due to digital transformation ~~becomes~~ less pronounced. Therefore, c~~CThe level of c~~orporate governance can be regarded as a substitute for~~has a substitution effect onwith~~ digital transformation. ~~Therefore, companies with poor governance can improve their capacity utilization through digital transformation, which can play a clear role advantage and contribute to improving social resource allocation.~~

~~c. The~~ Heterogeneity analysis based on industry

Columns (5) and (6) of Table 6 present~~give~~ the regression results for manufacturing and service firms, respectively. The~~ result is that the~~ estimated *DT* coefficient ~~of DT~~ for the sample of manufacturing firm~~ss sample~~ is significantly positive at the 5% level. The regression results for~~in~~ the sample of service firms, however,~~ show that while still positive in value, the results~~ are not significant, despite being positive. This~~It~~ suggests~~indicates~~ that, for manufacturing firms, the digital transformation ~~of enterprises~~ can~~, for manufacturing companies,~~ contribute to increased~~ athe rise in~~ capacity utilization through corporate innovation and improved production specialization~~ in production~~. In~~ByIn~~ contrast, the role of~~ service companies in using~~ digital transformation in improving~~to improve the~~ overcapacity ~~situation~~ of service firms is not significant. The most likely reason for this is that

Formatted: Indent: First line: 2.7 ch, Line spacing: Double

Formatted: Indent: First line: 0 ch

Formatted: Font: (Default) Times New Roman, 14 pt, Bold

Formatted: Line spacing: Double

Formatted: Font: (Default) Times New Roman, 12 pt

Formatted: Indent: First line: 2.36 ch, Line spacing: Double

Formatted: Font: (Default) Times New Roman, 12 pt, Italic

Formatted: Font: (Default) Times New Roman, 12 pt

th~~ose~~e manufacturing companies have significant~~manya lot of~~ over-capacities and ~~a lot of~~ potential~~s~~ to improve capacity utilization. Service firms, however,~~In contrast, compared to the manufacturing sector, service sector firms~~ have lower~~relatively small~~ transaction information friction and~~,~~ ~~hence,~~ higher capacity utilization, making the impact~~and the role~~ of digital transformation ~~is~~ less pronounced.

Table 6.~~–~~

Regression results of mechanism analysis

	(1) CU	(2) CU	(3) CU	(4) CU	(5) CU	(6) CU
DT	-0.364	0.219***	0.187	0.484**	0.151**	0.175
	(0.312)	(0.047)	(0.308)	(0.217)	(0.075)	(0.438)
size	-2.238***	-.473***	-1.312***	-.914***	-.711***	-1.913***
	(0.104)	(0.022)	(0.101)	(0.091)	(0.025)	(0.169)
roa	2.225**	0.539***	3.903***	1.194***	1.355***	3.843***
	(1.074)	(.09)	(.965)	(.455)	(.137)	(1.489)
lev	1.949***	.835***	2.58***	2.324***	1.223***	5.608***
	(0.529)	(0.087)	(0.491)	(0.407)	(0.108)	(0.896)
age	-0.229	0.062**	-0.227	-0.506***	-0.012	-0.257
	(0.217)	(0.029)	(0.208)	(0.138)	(0.036)	(0.313)
soe	-0.063	-0.162**	-0.095	-0.143	-0.092	-0.105
	(0.323)	(0.079)	(0.292)	(0.486)	(0.079)	(0.694)
Constants	21.146***	3.943***	11.985***	7.992***	6.192***	15.614***
	(1.024)	(0.146)	(0.918)	(0.667)	(0.193)	(1.344)
Observations	7844	7830	8125	7411	8629	4234
Adj-R^2	0.844	0.856	0.765	0.591	0.712	0.819

~~VI.~~ Discussion~~Conclusions~~

Overcapacity has resulted in significant~~led to serious~~ resource misallocation, and waste remains a major issue ~~problem~~ for economic growth that must be addressed ~~needs to be overcome~~. Most previous studies on~~Regarding~~ the path and methods to enhance capacity utilization have been conducted~~, most of the existing literature has~~

Formatted: Indent: First line: 2.7 ch

Formatted: Font: (Default) Times New Roman, 12 pt, Not Highlight

Formatted: Font: (Default) Times New Roman, 12 pt, Bold, Not Highlight

Formatted: Indent: First line: 0 ch

Formatted: Font: 12 pt, Bold, Not Highlight

Formatted: Font: (Default) Times New Roman, 12 pt, Bold, Not Highlight

Formatted: Font: (Default) Times New Roman, 18 pt

Formatted: Line spacing: Double

Formatted: Font: (Default) Times New Roman, 12 pt

Formatted: Indent: First line: 2.7 ch, Line spacing: Double

been studied from a macro perspective, such as government control, business environment, state-owned enterpriseSOEa soft budgets of SOEs, and market-oriented mechanisms, seldom investigatingwhile the role of digital transformation at an for enterprise levels is lacking. TheWith the advent of the digital era, the deep integration of real economies and digitalization has become athe trend and an inevitable result of future development since the advent of the digital era. The healthyHThe healthy development of the digital economy promotesis conducive to promoting the construction of a modern economic system. As a new type of production factor, data has a significant impact on changes in traditional production methods. The With high innovation, strong penetration, and wide coverage, the digital economy is not only a new economic growth point,, but also a fulcrum for transforming and upgrading traditional industries, and can be an important engine for building a modernized economic system, with high innovation, strong penetration, and widespread coverage. In this regard, While the existing literature has focused on the importance of digital transformation, however, no systematic study has been conductedfound thusso far on how digital transformations of enterprises improve overcapacity, and few studies have extensivelylittle literature has examined in depth the alternative roles of digital transformation and corporate governance.

Unlike previousexisting studies that investigated explore the governance of capacity utilization governance from macro and regional perspectives, this studypaper examines the impact of digital transformation on capacity utilization from an enterprise-level micro perspectivebyfrom micro-strategic enterprises. This study

Formatted: Indent: First line: 2.7 ch, Line spacing: Double

elucidates~~First, it analyzes~~ the theoretical mechanism by which the characteristics of digital transformation affect enterprise innovation and resource allocation efficiency;~~,~~ in other words~~that is~~~~i.e.,~~, how enterprise~~the~~ digital transformation ~~of enterprises~~ can effectively promote enterprise technological innovation, improve resource allocation efficiency, strengthen the firm's production specialization ~~their specialized production capacity~~, and thus effectively alleviate overcapacity. Furthermore~~Second~~, this study's findings demonstrate ~~the survey data of A-share listed enterprises using text analysis were~~~~is used to test the theoretical hypotheses. The test shows~~ that the effect of digital transformation on capacity utilization is inverted U-shaped, and that ~~the effect of~~ relying solely on ~~promoting enterprises'~~ digital transformation to suppress enterprise overcapacity is ineffective~~unsatisfactory~~. The~~Heterogeneity analysis finds that: the~~ impact~~effect~~ of digital transformation on capacity utilization is found to be more significant for enterprises with smaller capital ~~scales~~ and poorer governance.~~;~~ Moreover~~the~~, due to the lower likelihood of information mismatch in service firms, ~~less effective~~ digital transformation appears to alleviate ~~alleviates~~ overcapacity more effectively in~~relative to~~ manufacturing enterprises than in service ~~based~~ organizations~~industries~~ ~~because~~~~due of~~~~to the smaller chance of information mismatch~~.

It should be pointed out that although this paper uses the text analysis method of machine learning to carry out the overall situation of enterprise digital transformation, it has not yet achieved an accurate depiction of the digital aspects of the enterprise (such as research and development, production, sales, etc.). It is worth further research and exploration in the future to further characterize the digital

Formatted: Line spacing: Double

transformation of each link in the enterprise, to investigate more carefully the impact of the mechanism of each link's digital transformation on the external transaction costs and internal control costs of the enterprise.

Acknowledgements: I want to thank Shi Junwei, Liu Xinglin, Hu Shan and Guo Yanbing. I also want to thank the Center for Industrial Economics.

References

1. Bertani, F., Ponta, L., Raberto, M., Teglio, A., & Cincotti, S. (2021). The complexity of the intangible digital economy: an agent-based model. Journal of business research, 129, 527-540. https://doi.org/10.1016/j.jbusres.2020.03.041
2. Marini, G., & Pannone, A. (2007). Capital and capacity utilization revisited: A theory for ICT-assisted production systems. Structural Change and Economic Dynamics, 18(2), 231-248. https://doi.org/10.1016/j.strueco.2007.01.001
3. Dana Jr, J. D., & Orlov, E. (2014). Internet penetration and capacity utilization in the US airline industry. American Economic Journal: Microeconomics, 6(4), 106-37. https://doi.org/10.1257/mic.6.4.106
4. Teryima, S. J., & Sunday, A. (2015). The role of information communication technology (ICT) in enhancing productivity in local government administration in Benue State, Nigeria. International Journal of Business and Economic Development (IJBED), 3(1), 110-124
5. Spence, M. (1976). Informational aspects of market structure: An introduction. The Quarterly Journal of Economics, 591-597. https://doi.org/10.2307/1885323
6. Salop, S. C. (1979). Monopolistic competition with outside goods. The Bell Journal of Economics, 141-156. https://doi.org/10.2307/3003323
7. von Weizsacker, C. C. (1980). A welfare analysis of barriers to entry. The bell journal of economics, 399-420. https://doi.org/10.2307/3003371
8. Mathis, S., & Koscianski, J. (1997). Excess capacity as a barrier to entry in the US titanium industry. International Journal of Industrial Organization, 15(2), 263-281. https://doi.org/10.1016/0167-7187(95)01003-3
9. Spence, A. M. (1977). Entry, capacity, investment and oligopolistic pricing. The Bell Journal of Economics, 534-544. https://doi.org/10.2307/3003302
10. Ghemawat, P. (1984). Capacity expansion in the titanium dioxide industry. The Journal of Industrial Economics, 145-163.

Formatted: Font: (Default) Times New Roman, 12 pt

Formatted: Font: (Default) Times New Roman, 16 pt, Bold

Formatted: Font: (Default) Times New Roman, 16 pt, Bold

Formatted: Line spacing: Double

Formatted: Font: (Default) Times New Roman, 16 pt, Bold

https://doi.org/10.2307/2098506

11. Kirman, W. I., & Masson, R. T. (1986). Capacity signals and entry deterrence. International journal of industrial organization, 4(1), 25-42. https://doi.org/10.1016/0167-7187(86)90045-7

12. Mathis, S., & Koscianski, J. (1997). Excess capacity as a barrier to entry in the US titanium industry. International Journal of Industrial Organization, 15(2), 263-281. https://doi.org/10.1016/0167-7187(95)01003-3

13. Holmström, J. (2018). Recombination in digital innovation: Challenges, opportunities, and the importance of a theoretical framework. Information and organization, 28(2), 107-110. https://doi.org/10.1016/j.infoandorg.2018.04.002

14. Yoo, Y., Henfridsson, O., & Lyytinen, K. (2010). Research commentary—the new organizing logic of digital innovation: an agenda for information systems research. Information systems research, 21(4), 724-735.

15. Vargo, S. L., & Lusch, R. F. (2016). Institutions and axioms: an extension and update of service-dominant logic. Journal of the Academy of marketing Science, 44(1), 5-23. https://doi.org/10.1007/s11747-015-0456-3

16. Huang, J., Henfridsson, O., Liu, M. J., & Newell, S. (2017). Growing on steroids: Rapidly scaling the user base of digital ventures through digital innovation. MIS quarterly, 41(1). 10.25300/MISQ/2017/41.1.16

17. Abouzeedan, A., Klofsten, M., & Hedner, T. (2013). Internetization management as a facilitator for managing innovation in high-technology smaller firms. Global Business Review, 14(1), 121-136. https://doi.org/10.1177/0972150912466462

18. Reim, W., Parida, V., & Örtqvist, D. (2015). Product–Service Systems (PSS) business models and tactics–a systematic literature review. Journal of Cleaner Production, 97, 61-75. https://doi.org/10.1016/j.jclepro.2014.07.003

19. Tan, K. H., Zhan, Y., Ji, G., Ye, F., & Chang, C. (2015). Harvesting big data to enhance supply chain innovation capabilities: An analytic infrastructure based on deduction graph. International Journal of Production Economics, 165, 223-233. https://doi.org/10.1016/j.ijpe.2014.12.034

20. Porter, M. E., & Heppelmann, J. E. (2015). How smart, connected products are transforming companies. Harvard business review, 93(10), 96-114.

21. YANG De-ming; LIU Yong-wen. Why Can Internet Plus Increase Performance,[J]China Industrial Economics,2018(05):80-98. https://doi.org/10.19581/j.cnki.ciejournal.2021.09.007

22. Adam Smith. The Wealth of Nations[M]. Chicago: Univexrsity of Chicago Press, 1776

23. Eisenhardt, K. M. (1989). Making fast strategic decisions in high-velocity environments. Academy of Management journal, 32(3), 543-576.

24. Crozet, M., & Milet, E. (2017). Should everybody be in services? The effect of servitization on manufacturing firm performance. Journal of Economics & Management Strategy, 26(4), 820-841. https://doi.org/10.1111/jems.12211

25. Peng, G. C. A. (2013). Green ICT: A strategy for sustainable development of China's electronic information industry. China: An International Journal, 11(3), 68-86.

26. Lange, S., Pohl, J., & Santarius, T. (2020). Digitalization and energy consumption. Does ICT reduce energy demand?. Ecological Economics, 176, 106760. https://doi.org/10.1016/j.ecolecon.2020.106760

27. Yang, Deming, Liu, & Yongwen, 2018 Why Can Internet Plus Increase Performance,[J]China Industrial Economics, (05):80-98. https://doi.org/10.19581/j.cnki.ciejournal.2018.05.005

28. Adelman, M. A. (1955). Concept and statistical measurement of vertical integration. In Business concentration and price policy (pp. 281-330). Princeton University Press.

29. Buzzell, R. D. (1983). Is vertical integration profitable. Harv. Bus. Rev.;(United States), 61(1).

30. Fan Ziying, & Peng Fei, 2017 The Effects of "Business Tax Replaced with VAT Reform"on Firms' Tax Cuts and Industrial Division Based on the Perspective of Industrial Interconnection,[J]Economic Research Journal,52(02):82-95. CNKI:SUN:JJYJ.0.2017-02-007

31. Zhang, Y..Vertical Specialization of Firms: Evidence from China's Manufacturing Sector.[R]. CCER Series Paper, 2004.

32. Bing-Zhan Shi & Jian-Tong Li, 2020 Does the Internet Promote Division of Labor? Evidence from Chinese Manufacuring Enterprises,[J].Journal of Management World,36(04):130-149. https://doi.org/10.19744/j.cnki.11-1235/f.2020.0058

34. Wen & Ye, 2014 Analyses of Mediating Effects:The Development of Methods and Models,[J]Advances in Psychological Science,22(05):731-745. 10.3724/SP.J.1042.2014.00731

35. HUANG Qun-hui; YU Yong-ze; ZHANG Song-lin. Internet Development and Productivity Growth in Manufacturing Industry:Internal Mechanism and China Experiences,[J]China Industrial Economics,2019(08):5-23. doi:10.19581/j.cnki.ciejournal.2019.08.001.

Dear reviewers,

Thank you so much for your comments and professional advice. These comments helped to improve the academics of my article. Following your suggestions and requests, we have made corrections to the revised manuscript. At the same time, the grammar of the manuscript has been reviewed and edited. I hope our work can be improved again. Moreover, we would like to show the details as follows:

Reviewer 1＃

1- There are a huge number of published studies available in the field. How this study significantly contributes to the literature?

The author's answer: The reviewer makes a valid point that there are numerous articles on digital transformation and a relatively large number of articles studying capacity, but almost no articles examining the effect of digital transformation on capacity utilization. Therefore, this study opens up a contemporary research perspective on digital transformation. Moreover, most of the studies on digital transformation have been conducted from a positive perspective to investigate its impact mechanisms. Based on this, the research presented in this paper adds a study on the negative effects of digital transformation to fill the gap in research thinking. In addition, current measurements and characterization of capacity utilization are usually based on a macro level, e.g., country, region, etc., whereas the calculations of capacity utilization in this paper are at a more microscopic level, which leads to more accurate conclusions. (See *I. Introduction* in the revised manuscript for details)

2- Please cite papers from the different areas, as the author mainly focuses on Chinese literature such as "China has made significant achievements in economic growth". While many countries, such as India, the USA, and Israel, are adopting digital transformation.

The author's answer: The reviewer's comments are professional. With the development of science and technology, digital transformation is advocated in many countries, and there are many geographically distributed companies actively implementing digital management and services. However, there are some fundamental differences between these countries and regions and Chinese companies, such as over-building, over-investment, which hindered the efficient use of their resources and even prevents healthy economic development, and low capacity utilization and over-capacity. On top of this, the Chinese government urgently needs to find a tool to reverse this passive situation, so the digital transformation of local companies is particularly important. For these reasons, it is a more appropriate choice to study the impact of digital transformation on capacity ratios by analyzing the mechanism of its effect using data from Chinese-listed companies.

3- Expand the introduction by supporting the difference between digital and conventional transformation and cite some high-quality papers to support the idea.

The author's answer: Your advice is perfectly professional. Based on your comments, I have

added a high-level article related to the topic in the Introduction section as appropriate, as well as a more in-depth analysis and categorization of the current research on digital transformation. I have identified the current research gap in the analysis of the role of digitalization mechanisms and explored them as a research focus.

4- Scientific literature is lacking, please include more references related to the study.

The author’s answer: Thank you for your corrections. It has been added.

5- There are many characteristics of the overcapacity industry, but I can see only three have been mentioned in section II.

The author’s answer: Thank you for the correction. Excess capacity may exist at different stages of industry development. However, the causes of overcapacity are very different. For example, overcapacity is caused by competition in the early stages of industry development, or overcapacity is caused by declining market demand in sunset industries. Since this is not the focus of this paper, I have not highlighted it in the paper after further consideration.

6- Section II, First Paragraph, what do the distinctive Chinese characteristic please specify, and explain how these characteristics result in overcapacity.

The author’s answer: Thank you for the reminder, and based on your suggestion, I have added an introduction to Chinese firm features in the first section. The term capacity utilization, which serves as the focal explanatory variable in this study, has not been prominent in other country studies. This may be related to the historical process of overcapacity formation. It is well known that the most direct cause affecting capacity utilization is over-investment, and there are many causes that induce over-investment. In compiling the literature, I found that the main factors contributing to overcapacity in academia are broadly divided into three categories: 1. Industry competition, excessive competition in the industry, competitive strategies adopted by enterprises to deter and threaten their competitors, which triggers the expansion of production capacity and induces overcapacity; 2. Development dynamics, the frequent occurrence of investment surges in developing countries, and the role of market interest rates and price adjustment mechanisms are emphasized in the theory. The theory is that in developing countries, a large influx of capital into certain industries, i.e., an "investment boom", triggers the phenomenon of increasing overcapacity; 3. Government-business relationship, the most commonly studied theory in China, argues that local officials want to provide land and financing investments to local enterprises in the promotion race, resulting in the over-expansion of capacity and causing under-utilization of capacity.

7- Section II first paragraph, “The 2022 Government work report” cite the reference.

The author’s answer: Sorry for this error due to my negligence. Thank you for pointing it out, it has been corrected.

8- Please include the references to support your result and conclusion. Interpretation needs to be simplified and discussed with references.

The author’s answer: Thank you so much for your suggestion. I have made changes.

9- Include the limitation of the study.

The author's answer: In light of your comments and suggestions, we have added the limitations of the study in the "1 Introduction" section.

10- There are many typing and grammatical errors, which needs to be corrected.

The author's answer: Thank you for your advice. The grammatical problems have been corrected one by one.

Reviewer 2＃

Main comments

1. The English writing of this article is not up to standard, there are many grammatical errors, and the expression is not authentic, which is far from meeting the requirements of publication. There are unnecessary long difficult sentences in the article, and too many "and" are inappropriately used. I suggest the author(s) carefully revise the paper and submit it to a Chinese journal; otherwise the language should be greatly improved.

The author's answer: Thank you for your criticism. I re-read the article carefully and revised it, and have made sufficient repairs to eliminate grammatical errors, correct the use of "and", and make changes to the long and difficult sentences.

2. Many of the discussions and conclusions in this paper are neither supported by theoretical literature nor supported by theoretical model analysis, which greatly affects the credibility of this paper, e.g. the paper concludes that "digital transformation of enterprise will promote innovation, improve resource allocation and production capacity, so as to effectively curb overcapacity". How to curb overcapacity through digital transformation is an interesting question, but there is no model or theoretical analysis in this paper, so the mechanism is not well explored. As a result,many conclusions are just like basic judgments. I suggest the authors write a model to explain how digital transformation can better curb overcapacity compared with other ways.

The author's answer: The reviewer makes very good points and your suggestions are very professional. The logic and theory of the article have been further refined and reconstructed with your suggestions. In the introduction section, I appropriately introduce the many high-quality works in the literature to sort out the objective factors the influence capacity utilization at a more specific level, as well as to point out the reasons for selecting Chinese-listed companies as the target of the study. In addition, in sorting out the impact and mechanism of action of current enterprise capacity utilization, I discovered its research gap - on capacity utilization, which provides ideas for further analysis and paves the way for the theoretical analysis and research hypothesis in the later sections.

In the theoretical analysis sections, I adopt the previous analysis framework and introduce the main assumptions of this paper based on reading a large amount of high-level

literature. The theoretical analysis is taken from the classical literature, which enhances the credibility of the article. In the analysis of the impact of the digital transformation of firms on capacity utilization, two mediating variables, firm innovation, and specialized production, are introduced in this paper. Among them, for the hypothesis that digital transformation drives enterprise innovation, I explore in more depth the specific paths and implementation capabilities of innovation affecting enterprise capacity by specifically analyzing the factors that affect the role of capacity utilization and enumerating the mechanisms of impact of digital transformation. I think this can clearly show the mechanism of enterprise innovation as a mechanism. In the analysis of the specialization and division of labor and the innovative and important intermediary of specialized production of enterprises, I have explored it from the perspective of enterprise expansion and development. I am aware of the importance of clarifying the law of firm development for the study of this paper. Therefore, by reviewing the literature, I found that the horizontal expansion of enterprises inevitably promotes the efficient use of resources. Moreover, digital transformation can expand the production boundaries of enterprises and enhance specialized production capacity by reducing production costs and management costs. These factors can improve the efficiency of resource utilization, which in turn has a positive effect and impact on capacity utilization. Therefore, the specialized production segmentation of enterprises is presented in this section as one of the highlights of this study.

With these in-depth explorations and studies, the first hypothesis – the positive impact of digital transformation on enterprises – is presented here in this study. The theoretical framework is reconstructed to make the logic of the paper seem more reasonable and to make the theory of the paper more accessible and acceptable. (See I. Introduction and II. Theoretical analysis in the revised version for more details)

3. Three hypotheses are not well organized. As far as I'm concerned, H1, H2 and H3 are about a same thing: how digitalization promotes capacity utilization, so the main topic can be summarized into one hypothesis, which can be treated as the paper's main proposition and contribution. As stated in point 2, the authors can write a theoretical model to see whether the U-shaped nonlinear relationship can be derived from the model.

The author's answer: We would like to thank the reviewers for their very professional and pertinent suggestions. Following the suggestions of the reviewers, after further deliberation and careful consideration, I have made the following changes to the hypothesis section of the paper – Hypothesis 1 and Hypothesis 3 are combined, as Hypothesis 2 is retained as a research innovation of this paper.

The specific reasons are as follows: While these assumptions are all about the impact of digital transformation on capacity utilization, there are positive and negative mechanisms of action. A careful study of the literature reveals that the mechanism of action of digital transformation on capacity utilization has not only mediated the positive effects of innovation and specialized production, but also some negative adverse effects may exist. There is no guarantee that digital transformation will necessarily lead to efficient resource utilization. In the case of the communications industry, for example, the overuse of digital services has a more significant negative impact on data-intensive manufacturing. The lack of a better institutional design for advancing digital transformation in the initial stages of

digital transformation will lead to a failure to truly improve the efficiency of resource utilization in the enterprise. However, contemporary research has paid little attention to the negative impact of digital transformation on business development, that is, almost all research has focused on the positive impact of digital transformation on business. Therefore, the U-shaped character of the impact of digital transformation on the capacity utilization of firms can be used as an innovative analysis for the paper's research.

I strongly endorse the professional suggestions of the reviewers. Indeed, at the beginning of this research, I also considered whether to construct a theoretical model to explain this mechanism. By referring to a large body of literature and comparing analyses, I found that constructing a theoretical model does not explain the role of digital transformation as well as a theoretical analysis does, nor does it visually demonstrate the impact of digitalization in the enterprise. In general, the mechanism of digital transformation can be more generally understood and described by theoretical analysis, and therefore, after repeated consideration, I have chosen theoretical analysis to describe this mechanism of influence.

Minor comments and typos

1. The authors cite so many Chinese journals, and there are no more than 8 articles cited from international authoritative journals.

The author's answer: This has been increased to 28 articles.

2. The citing format in the article is wrong.

The author's answer: Thank you for the kind corrections. They have been corrected.

3. The formula is not fully displayed, although I can guess what it is.

The author's answer: I am very sorry for this error, which was due to my oversight. I thank the reviewers for their corrections and have made adjustments to confirm that it is fully displayed.

Thank you for giving us the opportunity to submit a revised draft of this manuscript for publication in Plos One. We appreciate the time and effort that you and the reviewers have devoted to providing feedback on our manuscript and are grateful for insightful comments and valuable improvements to our manuscript. We have incorporated most of the suggestions made by the reviewers. These changes are highlighted in the manuscript.

According to the reviewer's comments, we have revised the manuscript extensively. If there are any other modifications we could make, we would like very much to modify them and we appreciate your help. We hope that our manuscript will be considered for publication in your journal. Thank you so much for your help.

Yours sincerely,

Ning Zhao

November 20, 2022

Zhongnan University of Economics and Law
