# Supplementary material for: Impact of enterprise digital transformation on capacity utilization: Evidence from China
Source: PLoS One. 2023 Mar 17;18(3):e0283249. doi: 10.1371/journal.pone.0283249 (PMC10022773; doi:10.1371/journal.pone.0283249)
Supplement: Attachment — Submitted filename: Response to Reviewers.docx [file pone.0283249.s005.docx]

# Response to Reviewers

Dear reviewers:

Thank you for your letter and for the reviewers' comments concerning our manuscript. Those comments are all valuable and very helpful for revising and improving our paper,as well as the important guiding significance to our researches. We have studied comments carefully and have made correction which we hope meet with approval. Revised portion are marked in the paper. The main corrections in the paper and the responds to the reviewer's comments are as flowing: Responds to the reviewer's comments:

**Reviewer #1:** Please include the detailed limitation of studies, I can see only one point in last paragraph. Please describe and include all the limitation.
Thanks

**The author’s answer:** Thank you very much for your professional advice, which has helped me a lot in my research. According to your suggestion, I have supplemented the description of research limitations (see I. Introduction for details). Thank you again for your guidance on this article, which has inspired my research and writing.

**Reviewer #2:** The author has revised and improved the draft according to the comments of the first review, but there are still several following aspects that need further revisions:
**1.** There are still grammatical errors in the abstract, introduction, theory and hypotheses, discussion part of the revised version, which limit the clearness, coherence and readability of this paper. I revised the manuscript directly in the text sentence by sentence. Please refer to the yellow mark on pages 46-82 of the attached PDF file;

**The author’s answer:** Thank you very much for your professional and meticulous attitude. Your careful and responsible revision has motivated me to do research. Under your revision, I have re-read and modified the grammar of the full text in order to reach the standard. The article retains your professional and exquisite guidance. Thank you for your continued contributions to this manuscript, and I will work with absolute seriousness to improve the content of this paper.

**2.** The formulas are not successfully displayed, so I can't judge whether the formulas are correct and reasonable;

**The author’s answer:** I am very sorry for the situation. When I submitted the manuscript, I specifically checked the formulas and made sure they could be shown. Is it a matter of version? The formulas are similar to the content of the first edition. No essential changes were made. Please rest assured that I will contact the editorial department to ensure that the formulas are properly presented.

**3.** the note "t-statistics adjusted for firm-level clustering are in parentheses; ***, **, and * indicate significance at the 1%, 5%, and 10% levels respectively" should be shown in Table 3, 4, 5, and 6;

**The author’s answer:** Thank you very much for your reminding, I have made the changes and made sure they are fully displayed.

**4.** Please reconfirm that the citation format and the reference format meet the requirements of the journal.

**The author’s answer:** Thank you for your kind correction. This section has been corrected as required by the format.

Thank you for giving us the opportunity to submit a revised draft of this manuscript for publication in Plos One. We appreciate the time and effort that you and the reviewers have devoted to providing feedback on our manuscript and are grateful for insightful comments and valuable improvements to our manuscript. We have incorporated most of the suggestions made by the reviewers. These changes are highlighted in the manuscript.

According to the reviewer’s comments, we have revised the manuscript extensively. If there are any other modifications we could make, we would like very much to modify them and we appreciate your help. We hope that our manuscript will be considered for publication in your journal. Thank you so much for your help.

Yours sincerely,

Ning Zhao

February 28, 2023

Zhongnan University of Economics and Law
